# Autoantibodies and Molecular Mimicry in Alphavirus Chronic Arthritis: A Systematic Review

**DOI:** 10.3390/pathogens15020152

**Published:** 2026-01-30

**Authors:** Nosipho Zanele Masoto, Felicity Jane Burt

**Affiliations:** 1 Pathogen Research Laboratory, Division of Virology, Faculty of Health Sciences, University of the Free State, Bloemfontein 9301, South Africa; nosiphomasoto@gmail.com; 2 Division of Virology, National Health Laboratory Service (NHLS), Faculty of Health Sciences, University of the Free State, Bloemfontein 9301, South Africa

**Keywords:** alphavirus, arthritis, autoantibodies, autoimmunity, molecular mimicry

## Abstract

Chronic arthritis following arthritogenic alphavirus infections presents symptoms resembling autoimmune rheumatic diseases, raising questions about the underlying mechanisms, including molecular mimicry and autoantibody production. This systematic review evaluated evidence supporting molecular mimicry and the potential role of autoantibodies as predictive biomarkers in alphavirus-induced chronic arthritis. A comprehensive search of PubMed, Scopus and Web of Science was conducted following PRISMA 2020 guidelines and PECO framework. Thirteen studies met the inclusion criteria: four computational studies assessing peptide homology between viral and human proteins, and nine clinical studies evaluating autoantibodies in chronic post-alphavirus arthritis. Computational analyses identified conserved alphavirus peptides with sequence and structural similarity to human proteins implicated in autoimmunity, supporting the hypothesis of molecular mimicry. However, most lacked experimental validation. Clinical studies showed variable detection of autoantibodies, rheumatoid factors, anti-cyclic citrullinated peptide, and antinuclear antibodies in chronic patients, though seropositivity rates were inconsistent and generally low. Only one study reported a significant association between autoantibody levels and disease chronicity. The findings suggest a potential autoimmune component in post-alphavirus arthritis driven by molecular mimicry, though current evidence remains inconclusive due to methodological heterogeneity and limited validation. Autoantibodies may contribute to pathogenesis but are not reliable predictors of chronicity. Future longitudinal studies with standardized assays and validation of computational findings in human models are needed.

## 1. Introduction

Alphaviruses are enveloped spherical viruses, containing a positive-sense ribonucleic acid (RNA) and a genome of approximately 11.5 kb, capped at the 5′ terminus and polyadenylated at the 3′ end [1]. Alphaviruses use mosquitoes as their primary transmission vector and are therefore geographically distributed in areas where competent vectors are present. However, this group of viruses can potentially adapt to new vector species, allowing them to spread to non-endemic areas [2,3]. Based on historical geographic distribution and disease manifestations, alphaviruses were divided into two groups: New-World alphaviruses, which are mainly neurotropic and associated with encephalitis, and Old-World alphaviruses associated with arthritis, also known as arthritogenic alphaviruses. Arthritogenic alphaviruses include Sindbis virus (SINV), chikungunya virus (CHIKV), o’nyong’nyong virus (ONNV), Semliki Forest virus (SFV), Mayaro virus (MAYV) and Ross River virus (RRV), which are widely distributed in Africa, Asia and Europe and are known to cause outbreaks in certain parts of the continents [4,5].

While Old-World alphaviruses are traditionally associated with arthritic disease, emerging evidence indicates that CHIKV and others can cross the blood-brain barrier and cause neurologic manifestations in neonates, the elderly, or immunocompromised individuals [6]. Reported manifestations include encephalitis and peripheral neuropathies such as Guillain-Barré Syndrome [7]. Neurological disease has been reported in approximately 9–16% of suspected or confirmed CHIKV cases in the community setting and in up to one third or more hospitalized patients [7]. Experimental studies have shown that MAYV can infect human brain cells and induce a neuroinflammatory response suggesting potential for neuroinvasion [8]. Alphavirus tissue tropism and pathogenesis are shaped by a complex interplay of viral and host factors, and immune responses. Viral envelope proteins, such as E1 and E2, mediate receptor recognition and membrane fusion, influencing which cell types are permissive to infection. Genome encoded factors, including nonstructural proteins and RNA elements, can modulate replication efficiency and counteract host antiviral defenses, shaping tissue specificity [9]. Host determinants, including age-dependent differences in neuronal susceptibility, expression of viral receptors, and genetic polymorphisms affecting immune pathways, further influence viral replication in neural cells [10]. Age-dependent neuronal susceptibility arises from variations in immune maturity, neuronal vulnerability, and blood-brain barrier integrity across developmental stages. In neonates, immature immunity, permeable barriers, and vulnerable neural progenitor cells facilitate viral entry and replication in the central nervous system, whereas in older individuals, immunosenescence and reduced regenerative capacity increase the risk of neurological involvement [11]. These observations highlight that alphavirus pathogenesis represents a continuum of tissue involvement rather than a strict dichotomy between Old- and New-world, emphasizing the interplay between viral genetics, host susceptibility, and immune responses in determining infection outcomes.

**Figure 1:**
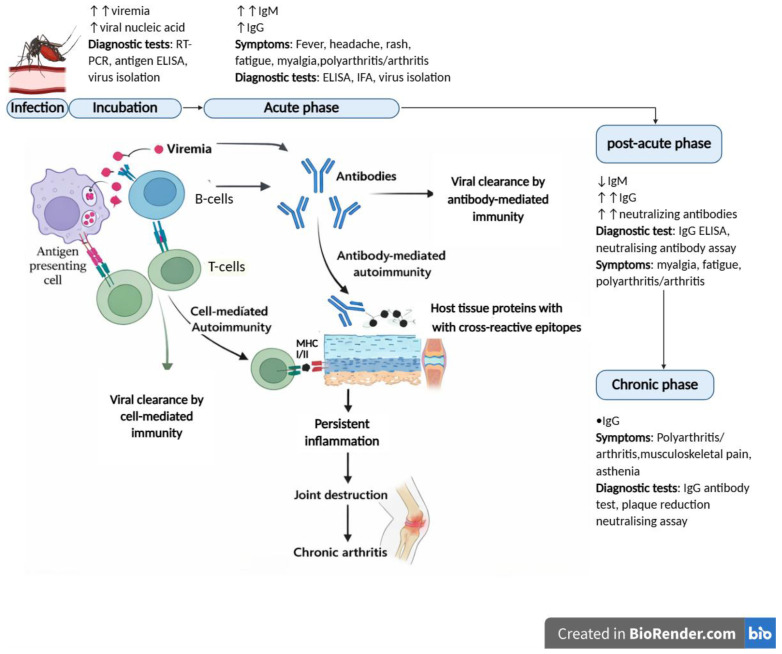
Proposed Autoimmune Mechanisms in Alphavirus-Induced Joint Disease. Following alphavirus infection, viral replication during the incubation and acute phases initiates innate immune activation, including antigen uptake by antigen-presenting cells. Viral antigens are processed and presented to virus-specific T-cells, leading to activation of adaptive immune responses and B-cell-mediated antibody production. In most individuals, coordinated antibody- and cell-mediated immunity results in effective viral clearance. However, in genetically susceptible hosts, viral peptide sharing structural or sequence homology with host joint proteins may be presented alongside self-antigens, enabling molecular mimicry and activation of cross-reactive T and B cells. These immune responses may promote autoantibody production, complement activation and sustained cytokine release, contributing to joint inflammation, tissue damage and progression from post-acute to chronic arthritis. The figure illustrates the temporal progression of disease phases and highlights the immune pathways and diagnostic approaches across the course of infection. ↑↑ Increase or high levels, ↑ gradual or delayed increase, ↓ decline, • stable levels over time. Reverse transcription polymerase reaction (RT-PCR), enzyme-linked immunosorbent assays (ELISA), Immunoglobulin M (IgM), Immunoglobulin G (IgG), Immunofluorescence assay (IFA). Created in https://BioRender.com.

Old-World alphavirus clinical manifestations are mild and self-limiting; the infection typically begins with an acute febrile illness characterized by fever, rash, and debilitating polyarthralgia. For most patients, symptoms are resolved within weeks. However, a subset of patients progresses to chronic arthritis marked by persistent joint pain, stiffness, and swelling that can last months to years after viral clearance. Due to their non-specific symptoms, alphavirus diagnosis relies on laboratory methods such as detection of viral nucleic acid using reverse transcription PCR (RT-PCR) during the acute phase of illness. However, since patients seldom present symptoms while viremic, confirmation most often depends on serological detection of IgG and IgM antibody responses [3,12] (Figure 1). While effective for confirming infection, these methods offer limited insight into the factors underlying clinical heterogeneity among patients, specifically why some individuals develop chronic disease while others recover fully.

One prominent hypothesis is that alphaviruses may induce autoimmunity via molecular mimicry. Molecular mimicry occurs when viral peptides share structural or sequence similarity with host proteins, leading to the generation of cross-reactive immune responses wherein the immune system continues to produce virus-specific antibodies and T-cell responses that cross-react with host autoantigens and potentially result in tissue-specific inflammation [13,14] (Figure 1). This mechanism has been proposed to underlie autoimmune diseases such as rheumatoid arthritis (RA), which shares clinical and pathological resemblance with alphavirus-induced chronic arthritis, including symmetrical joint involvement and prolonged inflammatory symptoms and autoimmune [15,16]. Supporting this hypothesis, several studies have reported the presence of autoantibodies, such as anti-cyclic citrullinated peptide (anti-CCP) antibodies and antinuclear antibodies (ANA), in patients with alphavirus-induced arthritis suggesting the presence of autoimmune mechanisms [17]. However, it remains unclear whether these antibodies indicate a true autoimmune process or merely represent secondary markers of ongoing inflammation. Therefore, this systematic review evaluated the existing literature on molecular mimicry as a potential trigger of autoimmunity in alphavirus arthritis and explored the potential role of autoantibodies in the pathogenesis and progression of disease, with a focus on their potential as predictive biomarkers of chronic disease.

## 2. Materials and Methods

The systematic review was conducted using Preferred Reporting Items for Systematic Reviews and Meta-analyses 2020 (PRISMA 2020) guidelines and PECO framework (Supplement S1). The protocol is registered in Open Science Framework (https://osf.io/az4wp/overview (accessed on 27 November 2025)). Ethics approval was obtained from University of the Free State, Environmental and Biosafety Research Ethics Committee (EBREC): UFS-ESD2023/0332.

### 2.1. Information Search Strategy

Search strategy was performed across three online databases: Scopus, Web of Science and PubMed, and search engines such as Google Scholar. Hand searching from the reference list of included studies was also performed to find papers not identified in the initial search. Medical Subject Headings (MeSH) terms were used to improve accuracy and enhance the reproducibility of the search. The following MeSH terms were used: alphavirus, molecular mimicry, autoantibodies, chronic arthritis and autoimmunity. The ”OR” operator was employed to combine search terms for related concepts and the “AND” operator to combine search terms for specific concepts. The complete search strategy and terms are found in Supplement S2.

### 2.2. The Inclusion and Exclusion Criteria

This study employed the PECO framework to guide the formulation of research questions (Supplement S1) and inclusion criteria. For the first question, we focused on arthritogenic alphaviruses (P), examining alphavirus peptides (E) in comparison to human proteins (C), with the outcome (O) being evidence of molecular mimicry through structural or molecular homology. For this question, both in silico and experimental studies, including animal models, were considered if they provided mechanistic validation of predicted viral-host homology. For the second question, the population (P) comprised of patients with chronic arthritis following alphavirus infection, with the exposure (E) being the presence of autoantibodies, compared to acute-phase patients, recovered individuals, or healthy controls (C). The outcome (O) was the prevalence or presence of autoantibodies in chronic patients, evaluated as potential biomarkers for virus-induced autoimmunity.

The inclusion criteria consisted of research papers that investigated alphavirus-induced chronic arthritis, papers that considered the level and/or the presence of autoantibodies in alphavirus-induced chronic arthritis, and studies with clear diagnosis or case definition of chronic alphavirus arthritis. in addition, studies investigating structural or molecular similarities between alphavirus peptides and human protein that could promote autoimmunity (in silico or experimental) were included. Only papers published in English were included. The exclusion criteria consisted of papers not published in English, duplicate publications, studies with a high risk of bias or poor quality of method, missing or unclear data and diagnostic methods, studies focusing only on acute infection and articles that did not follow the established PECO framework. Animal studies were excluded from analyses of autoantibodies but were retained when used to experimentally validate molecular mimicry prediction relevant to disease mechanism. For this systematic review, chronic patients were defined as patients who were diagnosed with any arthritogenic alphavirus using reliable laboratory tests, whether serological or PCR tests and had joint or musculoskeletal pain lasting for ≥3 months post-infection.

### 2.3. Quality Assessment and Data Extraction

The Rayyan tool for systematic reviews was used to remove duplicates and for the screening process (Rayyan|Home: https://rayyan.ai/users/sign_in) [18]. The search results were filtered using the inclusion and exclusion criteria. These were initially applied to titles and abstracts by two reviewers independently. Full reports were obtained for those studies that met the criteria. After screening, a standardized data extraction form was developed to extract relevant data from the included studies. Extracted data were organized into two categories: computational and mechanistic studies for molecular mimicry, and clinical studies for autoantibody detection and association with chronic arthritis. The risk of bias assessment was performed using the Joanna Briggs Institute (JBI) critical appraisal tools (Supplement S3) and National institute of Health (NIH) Quality Assessment Tool (Supplement S4). For computational studies, critical appraisal tools were developed with guidance from different sources summarized in Table 1 and detailed in Supplement S5.

### 2.4. Data Synthesis and Analyses

Due to the heterogeneity of the study design, findings were synthesized narratively. Computational and clinical evidence were analyzed separately followed by an integrated interpretation to explore mechanistic links between molecular mimicry and autoantibody-mediated autoimmunity. The focus was placed on identifying recurring homologous motifs and shared human targets as well as patterns of autoantibody response that could serve as predictive biomarkers for chronic arthritis.

## 3. Results

### 3.1. Study Selection

**Figure 2:**
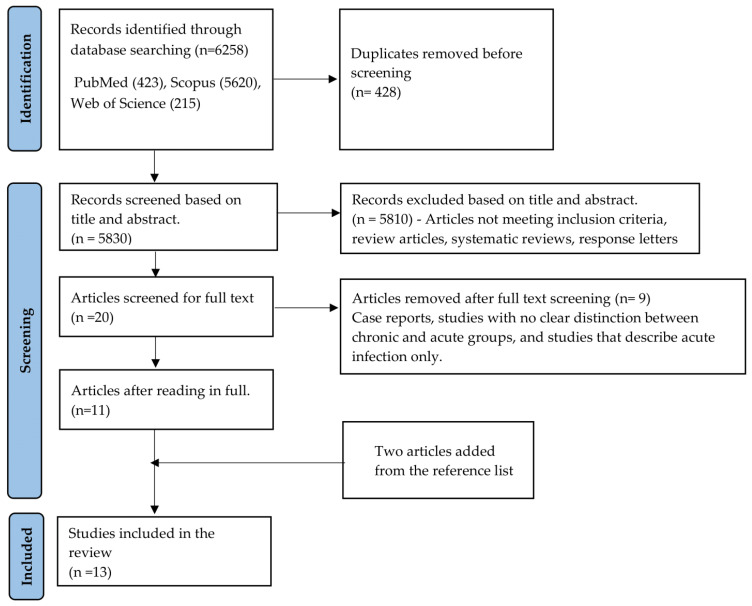
Flow chart detailing article selection for systematic review according to PRISMA guidelines [23].

The search strategy resulted in the identification of articles: 423 from PubMed, 5620 from Scopus, and 215 from Web of Science. In addition, two articles from references of the selected studies were included in the final evaluation. After screening, 13 articles fulfilled the inclusion criteria and were included in this review (Figure 2). Articles were excluded based on the title and abstract; exclusions included response letters, review articles, and systematic reviews. Several articles were also excluded after full-text review, and some are listed in Supplement S6.

### 3.2. Characteristics of Included Articles

The studies included in the analysis consisted of four computational studies and nine clinical studies. The computational studies explored the possibility of autoimmunity through molecular mimicry by investigating the existence of epitope or peptide homology using in silico analysis and bioinformatics tools [24,25,26,27]. The clinical studies investigated autoantibodies in alphavirus associated arthritis, focusing predominantly on CHIKV, with six conducted in India, two in France and one each in Mexico, Sudan, USA, China and Colombia (Table 2).

The definition of chronic disease was consistent across studies referring to the persistence of joint or musculoskeletal pain for three or more months following acute alphavirus infection in individuals with no prior history of RA or undefined arthritis. Joint pain was assessed either by a rheumatologist/clinician or reported via standard interviews.

Autoantibodies were evaluated at different stages of infection, in acute phase [28,29], chronic phase [17,30,31,32,33] and across sequential phases (acute, recovered and chronic phases) [34,35]. The control groups varied and included either healthy individuals [17,29,30], recovered patients [31,35], or patients without arthritis [28,33]. Two studies had no control groups [32,34]. All the articles reported on the presence or absence of autoantibodies in the chronic group. A few studies compared autoantibody levels between chronic and comparison groups [17,28,29,35] and only one study reported the association of autoantibodies with chronicity [29].

**Table 2:** Summary of studies reporting autoantibody detection in patients with chronic alphavirus-associated arthritis. Chronic cases were defined as joint or musculoskeletal symptoms persisting for ≥3 months following laboratory-confirmed alphavirus infection. Autoantibody positivity refers to the number of chronic cases testing positive within each study.

Study	Mean Age	Female: Male	Follow-Up (Months)	Sample Size (*n*)	Chronic Cases (*n*)	Autoantibody Positivity in Chronic Group (*n*)				Control Group (*n*)	Control Type
						RF	ANA	Anti-CCP	Others		
[28]	38.4 ± 20.9	1:1	6	32	23	8 (*p* = 0.002)		-	-	9	No joint pain
[17]	49.1 ± 10.2	3.78:1	22	34	34	7		None	-	10	Healthy controls
[30]	≥45	1:0.73	12	121 *	121	None	3	-	-	80	Healthy controls
[34]	All age groups	1:0.8	24	202 **	202	14	14	-	-	n/a	No controls
[31]	60.1 ± 1.4	2:1	156	30	17	3	3	3	6	13	Recovered
[32]	35 ± 14.1	1:1.15	10	203	94	None		1/20 ***	-	n/a	No controls
[29]	33.1 ± 14.7	1:1.49	24	167	49	cd ^#^		cd (*p* = 0.0003)	-	102	Healthy controls
[33]	All age groups	1:0.99	36	22 *	22	None	4	None	None	20	Without arthralgia
[35]	48 ± 15.0	1.5:1	12	10	2	2 (*p* < 0.01)		-	-	8	Resolved cases

* Included samples from patients with post-CHIKV arthralgia, ** included samples from patients with chronic arthritis, *** included 20 patients from the chronic group, cd ^#^ reported RF positive cases for all CHIKV infected patients, not chronic patients specifically, cannot determine (cd), not applicable (n/a), rheumatic factor (RF), antinuclear antibody (ANA), anti-citrullinated protein (anti-CCP).

### 3.3. Computational Studies Characteristics

Four computational studies investigated molecular mimicry between arthritogenic alphavirus antigens and human proteins. Two studies focused on CHIKV, while the other two studies targeted multiple viruses. Three studies examined homology between viral epitopes/peptides and human proteins using in silico analysis without cross-validation of results, while only one study validated its findings using animal models and clinical samples [26] (Table 3). All four computational studies identified conserved sequences or structural homology between alphavirus proteins and human proteins implicated in joint integrity and immune regulation, most commonly collagen II and complement C3. The potential consequences of mimicry of these proteins on the immune response are listed in Supplement S7.

### 3.4. Risk of Bias Assessment

The risk of bias for non-computational studies was assessed using JBI and NIH tools, while a custom appraisal checklist was developed for computational studies (Table 1, Supplement S5. Among the nine clinical studies, five exhibited a low risk of bias [17,28,29,32,35], while four demonstrated moderate risk due to confounding, high loss to follow-up and lack of strategies to address confounding [30,31,33,34] (Table 4). One study was excluded due to high risk of bias (Supplements S4 and S6).

For computational analyses, three studies had a moderate to high risk of bias due to the absence of validation through in vivo or in vitro analyses [24,25,27], and one showed low to moderate risk, supported by partial validation using animal models [26] (Table 5).

### 3.5. Summary of Findings

This section summarizes the main results of studies included in this review, providing a narrative synthesis of data presented in Table 2 and Table 3.

#### 3.5.1. Autoantibody Studies (Table 2)

Nine clinical investigations evaluated autoantibody response during and after alphavirus infection. Rheumatoid factor (RF), antinuclear antibodies (ANA) and anti-cyclic citrullinated peptides (anti-CCP) were the most frequently assessed markers. Long-term studies provided insight into these immune patterns. In a 13-year follow-up of patients with CHIKV infection, RF, ANA and anti-CCP were occasionally detected among individuals with persistent arthritis, although positive rates were substantially lower than those observed in established RA [31]. Another investigation reported low levels of ANA positivity in 20% of chronic CHIKV cases, with no other autoimmune markers detected [33]. Across several cohorts, RF positivity ranged from 13% to 34% among patients with chronic arthritis and was occasionally sustained up to 12 months after infection [28,29,35]. Some studies noted that RF levels remained elevated in patients with ongoing joint pain while declining in those who recovered [28,35]. Anti-CCP antibodies were inconsistently detected but occasionally correlated with the persistence of arthritis from acute to chronic phases [29]. In a 10-month follow-up, RF became undetectable in some chronic cases and anti-CCP was identified only in a patient with pre-existing joint disease before CHIKV infection [32] (Table 2). Most studies did not demonstrate a strong or reproducible association between autoantibody presence and chronic disease, suggesting that autoantibodies are indirect markers of immune activation rather than primary drivers of chronicity.

#### 3.5.2. Molecular Mimicry (Table 3)

Four in silico analyses investigated potential mimicry between alphavirus and human proteins. In one analysis, 24 conserved regions within polystructural proteins of six alphaviruses (CHIKV, RRV, SFV, ONV, BFV and MAYV) showed 52–100% sequence homology with human arthritis related proteins, several of which are known autoantibody targets in RA [27]. These regions displayed predicted HLA class II binding capacity and docking analysis revealed close structural alignment with the HLA-DRB1 allele. Another study identified two conserved immunodominant motifs within CHIKV E1 glycoprotein (Peptide A: 201 GDIQSRTPESKDVYANTQLV220 and Peptide B: 318 IKYAVSKKGKCAVHSMTNAV 338) that shared structural similarity with HLA-B27 and complement components C3 and C5 [26]. Immunization of mice with these peptides resulted in localized myositis-like inflammatory lesions, consistent with bioinformatic prediction of molecular mimicry. However, the study did not assess autoantibody production or systemic immune dysregulation, indicating that the observed pathology reflect peptide driven inflammatory response rather than definitive autoimmune response [26]. Further analysis of viral sequences demonstrated more than 60% homology between alphavirus peptides and human collagen II (CII), including motifs matching RA-associated DR alleles [25]. A total of 37 identical sequences were identified between CHIKV polyprotein and human arthritis- related proteins, overlapping known human immune-reactive epitopes [24]. Collectively, these computational studies revealed recurrent sequence similarities between alphavirus and human proteins linked to arthritis, although most lacked experimental validation (Table 3).

## 4. Discussion

Alphavirus infections are associated with chronic arthritis, raising questions about their potential to trigger autoimmune-like mechanisms through molecular mimicry and autoantibody production. The synthesis of computational data demonstrates that alphaviruses contain peptides exhibiting strong sequences and structural homology to host autoantigens. These molecular similarities support the biological plausibility of mimicry-driven autoimmunity but remain largely theoretical, as direct functional confirmation of cross-reactivity in humans is lacking.

Molecular mimicry occurs when viral or microbial peptides share structural or sequence similarity with host proteins, creating the potential for immune recognition of self-antigens [37]. Cross-reactivity arises when the adaptive immune system, particularly T cells or antibodies generated against the pathogen, mistakenly binds to these structurally similar host proteins. This functional immune response can result in tissue damage or chronic inflammation [38]. Importantly, molecular mimicry is a prerequisite for cross-reactivity, but not all mimicry leads to cross-reactivity, and not all cross-reactivity leads to autoimmune disease [39]. The clinical outcome depends on host factors such as genetic susceptibility, immune regulation, and the local tissue environment. In this way, molecular mimicry provides the structural basis for potential immune misrecognition, while cross-reactivity represents the actual execution of the autoimmune response, linking pathogen exposure to the development of chronic immune-mediated pathology [39]. The concept of molecular mimicry has gained significant attention in understanding the potential link between viral infections and autoimmune diseases, such as rheumatoid arthritis (RA).

### 4.1. Evidence of Molecular Mimicry Between Alphavirus Peptides and Human Proteins

Across the reviewed studies, alphaviruses including CHIKV, SFV, ONNV, BFV, and MAYV were shown to have conserved epitopes homologous to human proteins involved in joint integrity and immune regulation, notably CII and C3 [24,25,27]. These findings establish a structural foundation for potential cross-reactivity and imply that infection could trigger autoreactive immune responses. This mechanism has pathways similar to those observed in RA, where immune recognition of citrullinated CII peptides contributes to chronic joint inflammation and tissue destruction [40,41]. In patients with chronic arthritis following alphavirus infection, antibodies directed against CII have been detected in the serum, synovial fluid, and cartilage, further implicating CII-specific autoimmunity in disease development [41,42,43].

Likewise, complement activation via C3 and C5 promotes recruitment of inflammatory cells, amplification of cytokine cascades and cartilage degradation [44]. Clinically, RA patients exhibit significantly higher systemic concentrations of C3a and C5 compared with healthy controls and anti-CCP positive individuals without inflammatory arthritis, suggesting that complement activation, particularly via C3, may represent a critical transition point from preclinical autoimmunity to overt joint disease [45,46]. Evidence from alphavirus arthritis models supports this role. In RRV infection, mannan-binding lectin (MBL) deficient mice display reduced C3 deposition and joint damage, while C3 deficient mice exhibit markedly attenuated disease and tissue destruction, indicating that C3 is a key driver of alphavirus-induced inflammation [47,48]. However, whether this reflects mimicry-driven autoimmunity or secondary inflammation amplification remains uncertain.

Evidence from in vivo and in silico models partially supports this immunopathogenic potential. For instance, CHIKV E1 derived peptides (E1: 201–220 and E1: 318–338) homologous to human inflammatory proteins including C3 and HLA-B27, induced myositis-like inflammation in mice, particularly in those previously exposed to CHIKV [26]. Computational predictions further identified viral epitopes with homology to a major histocompatibility complex (MHC) class 1 that has a strong affinity for RA-associated HLA-DRB1 alleles [27], implying potential for autoreactive T-cell activation. HLA-DRB1 polymorphisms, particularly alleles encoding the shared epitope, are the most powerful genetic risk factors for RA and are known to influence both the selection and expansion of T-cell populations [49]. In RA, a gene-dose effect in which two risk alleles are inherited further increases disease risk and is linked to clonal expansion of autoreactive T cells, including cytotoxic CD4^+^CD28^−^ subsets implicated in joint and systemic pathology [49,50]. By analogy, individuals carrying HLA-DRB1 alleles capable of presenting viral peptides that mimic self-proteins may be genetically predisposed to misrecognize viral antigens, potentially linking molecular mimicry to both impaired antiviral immunity and initiation of autoimmune pathology in virus-induced arthritis.

Overall, these studies consistently suggest that alphaviruses harbor conserved motifs that resemble human immune proteins, supporting the role of molecular mimicry in promoting autoimmunity. However, most evidence remains largely bioinformatic, highlighting the need for further validation in human cohorts. Critical gaps remain in understanding how host genetics influence individual susceptibility and in delineating the immune pathways that lead from infection to chronic arthritis. Future research should focus on confirming molecular mimicry in humans diagnosed with alphavirus-induced arthritis, including whether human proteins with shared homology elicit similar pathogenic responses as the virus in animal models and whether antibodies against these homologous proteins are detectable in affected individuals. Such studies will clarify the mechanistic link between viral exposure and autoimmune arthritis.

### 4.2. Autoantibodies as Indirect Markers of Autoimmunity and Potential Predictive Biomarkers of Chronicity

The role of autoantibodies in alphavirus-induced arthritis remains incompletely defined. Studies investigating RF, anti-CCP and ANA following alphavirus infection show variable detection rates across cohorts [20,21,22,23,24,25,27]. RF and anti-CCP have been reported sporadically in patients with persistent joint symptoms, whereas ANA positivity is generally low and transient. The overall seropositivity in these studies is lower than that observed in established autoimmune diseases such as RA and systemic lupus [31,33], suggesting that autoantibody generation in alphavirus infection is an inconsistent and transient event rather than a hallmark of chronicity.

In a longitudinal study, RF levels remained elevated among patients with ongoing arthralgia but declined in those who recovered, suggesting a possible association between RF seropositivity and continued symptoms [28,35]. Similarly, anti-CCP antibodies occasionally correlate with prolonged arthritis from acute to chronic phase [29]. This indicates that certain autoantibody patterns may reflect disease persistence; however, their predictive value is weak due to inconsistent replication across studies. In addition, these findings suggest that alphavirus infection may transiently activate autoreactive B cells without sustaining full autoimmune propagation. Other studies found no significant differences in autoantibody prevalence between chronic and recovered individuals [30,32,33], further limiting their reliability as biomarkers for disease progression.

These sporadic detections of anti-CCP in chronic alphavirus patients may relate to the intense local inflammatory environment described in the context of molecular mimicry and complement activation. In alphavirus arthritis models, C3 is a key driver of synovial inflammation and tissue damage [47,48]. Persistent complement activation could promote citrullination of joint proteins, including CII, creating neoepitopes capable of triggering anti-CCP production in susceptible individuals [29,32,51]. Although such a pathway remains hypothetical, it aligns with patterns observed in RA, where excessive complement activity correlates with anti-CCP–positive disease [45,46]. Therefore, the presence of RF or anti-CCP in some chronic alphavirus cases may be a byproduct of prolonged inflammation rather than a primary autoimmune mechanism. The short-lived nature of these antibodies further supports the idea that alphavirus-induced arthritis is largely an autoimmune-like response driven by immune dysregulation secondary to infection rather than persistent loss of tolerance. To date, the potential relationship between complement activation and anti-CCP seropositivity in alphavirus infections remains largely unexplored.

### 4.3. Integrative Pathogenic Link Between Molecular Mimicry and Autoantibody Production

The relationship between molecular mimicry and autoantibody formation is likely to represent a continuous process rather than an isolated event and must be considered in the context of immune tolerance. Under normal conditions, central and peripheral tolerance mechanisms limit the activation of T and B cells, and even the presence of low affinity cross-reactive epitopes [52]. However, during alphavirus, strong innate immune activation, high levels of inflammatory cytokines and enhanced costimulatory signaling can temporarily override these safeguards. Presumably this occurs by creating an immunogenic environment in which viral peptides with homology to self-antigen may be presented to adaptive immune system, permitting activation of quiescent autoreactive lymphocytes [53]. Viral peptides exhibiting homology to CII and C3 could therefore initiate early immune activation by triggering antigen presenting cells and activating autoreactive T-cells in genetically susceptible hosts. Therefore, activating B-cells that produce cross-reactive antibodies, including RF or anti-CCP, particularly in an environment sustained by complements and cytokine release.

Following this initiation phase, complement-mediated inflammatory amplification may further shape disease outcomes. Complement fragments such as C3a and C5a amplify local inflammation, enhance antigen presentation and promote citrullination of self-proteins. These processes can mimic the immunopathology of RA, resulting in tissue-specific damage. However, unlike RA, alphavirus-induced responses appear self-limited, potentially due to efficient viral clearance or restoration of immune tolerance once the infection resolves. In contrast, individuals with genetic or immunologic susceptibility, such as those carrying specific HLA risk alleles, pre-existing subclinical autoimmunity or prolonged innate immune activation may fail to re-establish tolerance. This failure allows low level autoreactivity and persistent inflammation to evolve into chronic disease. Therefore, chronic alphavirus arthritis likely results from intersection of viral factors and host factors rather than from molecular mimicry alone.

### 4.4. Viral Persistence as a Complementary Mechanism

Although this review focused primarily on immune response and molecular mimicry, viral persistence has also been proposed as a contributing factor in alphavirus chronic arthritis. Support for this hypothesis includes reports of prolonged anti-IgM responses with anti-CHIKV IgM detected up to 13 years after infection, suggesting ongoing or intermittent antigenic stimulation [31]. In addition, CHIKV antigen has been detected in synovial macrophages and CHIKV RNA in synovial biopsy tissue 18 months after infection [16]. This raises the possibility that viral persistence within tissue resident immune cells may contribute to sustained inflammation and joint pathology.

In contrast, another study failed to detect evidence of viral persistence in chronic disease. A cross-sectional analysis of patients with CHIKV arthritis, followed over a 22 month period, found no detectable CHIKV RNA, infectious virus or evidence of viral proteins in plasma and/or synovial fluid [17]. Together, these contrasting observations suggest that viral persistence is unlikely to be a universal mechanism underlying alphavirus arthritis but may occur in selected individuals or within specific tissue compartments.

Importantly, viral persistence and immune-mediated mechanisms are not mutually exclusive. Low level or compartmentalized viral antigens may sustain innate immune activation, complement signaling or antigen presentation. This can lower immune tolerance thresholds and amplify molecular mimicry-driven autoreactive responses initiated during acute infection. In patients without detectable virus, persistent arthritis is more likely driven by immune dysregulation and autoimmune-like processes that persist after viral clearance. Clarifying the relative contribution of viral persistence and immune-mediated pathology will require longitudinal studies combining sensitive viral detection in tissue compartments with detailed immune profiling.

### 4.5. Limitations, Future Directions and Therapeutic Implications

Despite growing evidence, methodological heterogeneity across studies limits the strength of conclusions that can be drawn. Computational studies lack experimental verification of predicted homologies and clinical studies included in this review differ in several key methodological aspects, such as sample size, length of follow-up, disease stage at the time of blood collection, sample sources, and the types of control groups used. Additionally, certain studies often experienced significant loss to follow-up and biomarker analyses were performed on a limited number of patients, either due to small overall study populations or because only a subset of patients underwent more detailed laboratory testing. The methods used to detect each autoantibody also varied across studies. All these complicated cross-study comparisons. Moreover, while much of the current research has focused on CHIKV, greater attention should also be directed toward other arthritogenic alphaviruses to fully understand the broader spectrum of post-viral autoimmune risk and arthritis.

To clarify the role of molecular mimicry and immune dysregulation in alphavirus arthritis, future research should prioritize experimental validation of predicted epitopes through peptide binding and cross-reactivity assays in vitro or in vivo. Additionally, studies should focus on host genetic profiling to identify susceptibility patterns and multi-omics approaches to distinguish mimicry driven autoimmunity from non-specific inflammation. Understanding the potential autoimmunity triggered by alphavirus infections could provide valuable insights for the development of targeted therapeutic interventions. Existing immunology-based treatments for autoimmune diseases, such as biologics targeting TNF-α or IL-6, B cell depletion therapies, and peptide-based inhibitors designed to block pathogenic antibody-antigen interactions, offer potential frameworks for managing alphavirus-induced chronic arthritis. However, this systematic review did not examine therapeutic strategies, as this aspect was beyond the scope of the current study and was not captured in the search strategy. Nonetheless, research on potential therapies has been conducted and discussed in detail in other comprehensive reviews [54,55,56].

## 5. Conclusions

This systematic review demonstrates that arthritogenic alphaviruses can trigger immune responses that phenotypically resemble autoimmunity. Computational evidence supports molecular mimicry between viral peptides and host autoantigen, providing a plausible structural base for immune misrecognition that could lead to chronic inflammation. However, while these findings suggest potential for cross-reactive immune reactivation, there’s no evidence of antigen specific cross reactivity in humans, reflecting a major research gap rather than evidence against its occurrence. Autoantibodies including RF, anti-CCP and ANA are occasionally detected in chronic alphavirus-induced arthritis, but their presence is inconsistent indicating that they may represent transient immune activation rather than sustained autoreactivity. These patterns suggest that host genetic factors and immune regulatory mechanisms are likely to influence whether infection resolves or evolves into persistent joint inflammation.

## Figures and Tables

**Table 1:** Risk of Bias Assessment Guidelines for computational studies.

Bias Domain	Description	Low Risk	Moderate Risk	High Risk	Source Adapted from
Selection Bias	Representativeness of biological and computational data sources	Broad, justified selection of viral strains, host proteins, and datasets	Minor dataset limitations	Narrow or unjustified data selection	[19,20]
Input Data Preparation & Optimization	Quality and preprocessing of sequences, structures, and ligands	Fully optimized inputs with quality control	Partial optimization	No optimization or quality checks	-
Performance Bias	Appropriateness and standardization of computational tools	Validated tools with standardized parameters and cross-validation	Appropriate tools with inconsistent parameters	Unvalidated or outdated tools	JBI critical appraisal checklist for cohort studies
Target Selection	Quality and biological relevance of structural targets	High-resolution experimental structures or validated models	Validated homology models	Low-quality or unvalidated targets	[21]
Detection Bias	Validation of in silico findings	Supported by experimental or independent datasets	Limited validation	No validation performed	[21]
Reporting Bias	Transparency and completeness of reported findings	Positive and negative results reported	Partial reporting	Selective reporting of favorable results	[22]
External Validity	Reproducibility of computational findings	Fully reproducible methods	Partially reproducible	Non-reproducible workflows	[22]

Joanna Briggs Institute (JBI).

**Table 3:** In silico evidence of molecular mimicry between alphavirus peptides and human proteins.

Study	Targeted Pathogens	Pathogen Protein	Targeted Human Protein	Human Protein with Shared Homology	Results
[25]	SFV, MAYV, ONNV, CHIKV BFV, SINV	Envelope or capsid protein	Collagen II (CII 1168–1180)	Collagen II	Alphavirus-derived peptides showed significantly greater similarity to CII than random or non-alphavirus peptides, particularly in antigenicity and hydrophobicity profiles (*p* = 0.01 and *p* = 0.03), supporting potential immunological cross-reactivity.
[26]	CHIKV	E1 glycoprotein	Broad	Complement C3, Receptor protein tyrosine phosphate	Two conserved E1-derived peptides displayed homology with multiple human inflammatory proteins, including C3, and induced localized myositis-like inflammation in peptide-immunized mice, supporting biological plausibility of mimicry-driven inflammation.
[27]	SFV, MAYV ONNV, CHIKV, BFV, SINV	Structural protein	Broad	Collagen alpha-1(II), Complement C3, MHC class 1 polypeptide B	Twenty-four of 41 conserved alphavirus regions exhibited 52.7–100% sequence homology with human arthritis-associated proteins and showed predicted HLA class II binding capacity, suggesting potential for autoreactive immune activation.
[24]	CHIKV	Polyprotein	Proteins associated with arthritis	Complement C3, Collagen II, MHC class 1 polypeptide B, Tyrosine protein phosphate non-receptor	Thirty-seven identical sequences were identified between CHIKV polyprotein and human arthritis-related proteins, recurring across 103 experimentally immune-positive human epitopes, indicating extensive overlap with known immune-reactive regions.

Sindbis virus (SINV), chikungunya virus (CHIKV), o’nyong’nyong virus (ONNV), Semliki Forest virus (SFV), Mayaro virus (MAYV), Barmah Forest virus (BFV), envelope (E), major histocompatibility complex (MHC), human leukocyte antigen (HLA).

**Table 4:** Risk-of-bias assessment of included non-computational studies using JBI and NIH quality appraisal tools.

**JBI Critical Appraisal Checklist for Analytical Cross-Sectional Studies**				
JBI Criteria	[28]	[17]	[29]	[30]
Criteria for inclusion clearly defined?	✔	✔	✔	✔
Study subjects and the setting described in detail?	✔	✔	✔	✔
Exposure measured in a valid and reliable way?	✔	✔	✔	✔
Standard criteria used for measurement of the condition?	✔	✔	✔	✔
Confounding factors identified?	✔	✔	✔	✔
Strategies to deal with confounding factors	✔	✔	✔	✘
Outcomes measured in a valid and reliable way?	✔	✔	✔	✔
Appropriate statistical analysis	✔	✔	✔	✔
Risk of Bias	Low	Low	Low	Low to moderate
Overall Appraisal	Include	Include	Include	Include
**JBI Critical Appraisal Checklist for cohort studies**				
JBI Checklist Questions	[33]	[31]		[32]
Were the two groups similar and recruited from the same population?	✔	✔		✔
Were the exposures measured similarly to assigning people to both exposed and unexposed groups?	✔	✔		✔
Exposure measured validly and reliably?	✔	✔		✔
Confounding factors identified?	✘	✘		✔
Strategies to deal with confounding factors stated?	✘	✘		✔
Were the groups/participants free of the outcome at the start of the study (or at the moment of exposure)?	✔	X		✔
Were the outcomes measured in a valid and reliable way?	✔	✔		✔
Was the follow-up time reported and sufficient to be long enough for outcomes to occur?	✔	✔		✔
Was follow-up complete, and if not, were the reasons for loss to follow-up described and explored?	✘	✘		✔
Were strategies to address incomplete follow-up utilized?	✘	✘		N/A
Was an appropriate statistical analysis used?	✔	✔		✔
Risk of bias	Moderate	Moderate *		Low
Overall Appraisal	Include	Include		Include
**JBI Checklist for Case Series studies**				
JBI Criteria		[35]		[36]
Clear inclusion criteria?		✔		✔
Was the condition measured in a standard, reliable way for all participants?		✔		✔
Were valid methods used for the identification of the condition for all participants included in the case series		✔		✔
Did the case series have consecutive inclusion of participants?		✔		Unclear
Did the case series have complete inclusion of participants		Unclear		Unclear
Was there clear reporting of the demographics of the participants in the case series?		✔		unclear
Was there clear reporting of clinical information of the participants?		✔		✔
Were the outcomes or follow-up results of cases clearly reported?		✔		Unclear
Was there clear reporting of the presenting site(s)/clinic(s) demographic information?		✔		✘
Were statistical methods appropriate?		✔		✘
Risk of Bias		low		High
Overall appraisal		Include		Exclude
**NIH Assessment tool for before-after (Pre-Post) studies with no control group**				
Question				[34]
Was the study question clearly stated?				✔
Were eligibility/selection criteria for participants prespecified and clearly described?				✔
Were participants representative of the population?				✔
Were all eligible participants included?				✔
Was the sample size sufficiently large to provide confidence in findings?				✔
Was the intervention (exposure) clearly described?				✔
Were outcome measures pre-specified, valid, and reliable?				✔
Were outcome assessors blinded to exposure status?				NR
Was follow-up sufficiently long to detect an effect?				✔
Was loss to follow-up ≤20%?				CD
Were statistical tests appropriate for before-after comparisons?				✔
Were multiple outcome measures assessed before and after the exposure?				✔
Was there any attempt to minimize confounding variables?				✘
Were missing data handled appropriately?				✔
Risk of bias				Moderate
Overall Appraisal				Include

* Supplement S3, ✔ yes, ✘ no, not reported (NR), cannot determine (CD, not applicable (N/A), Joanna Briggs Institute (JBI), National Institutes of Health (NIH).

**Table 5:** Risk of bias assessment for computational studies.

Criteria	[27]	[24]	[26]	[25]
Selection Bias	Low Multiple viruses, viral proteins, and host proteins were analyzed.	Moderate Multiple human proteins associated with arthritis were analyzed, justified why the study focuses on one viral polyprotein. Bias: Focused on Indian Ocean Lineage CHIKV.	Low Multiple human proteins and multiple peptides of a protein were analyzed	Low Investigated multiple alphaviruses
Input Data Preparation & Optimization	Low MUSCLE is a validated alignment tool. Use BLAST and UniProt, manually curated protein using the Open Targets Platform server 38	Moderate Identified peptide matches but no ligand/structure refinement	Moderate Used sequence-based tools such as BLAST and ClustalW	Low The study used UniProt for reliable sequence data, ensuring high-quality inputs. MUSCLE was used for multiple sequence alignment.
Performance Bias (Computational Methods & Software Use)	Low Validated bioinformatics tools, including BLAST for homology search, ProPred II for T-cell epitope prediction, and IEDB/ElliPro for B-cell epitope prediction. Three different prediction algorithms (Bepipred, Emini Surface Accessibility, Kolaskar & Tongaonkar) were used.	Moderate Validated IEDB but no cross-validation.	Low Cross-validation was performed using multiple tools (IEDB, EMBOSS, COUDES) for epitope and beta-turn prediction. BioXGEM was used for structural similarity, complementing sequence-based predictions.)	Moderate BLAST was used for homology search, a well-validated tool. Used IEDB tools (ProPred II) for epitope prediction, which are widely accepted in immunoinformatics. Applied multiple methods for analyzing peptide binding properties (e.g., hydrophobicity, antigenicity). No cross-validation with alternative epitope prediction tools
Target Selection (For Molecular Docking Studies)	Moderate GalaxyPepDock was used for peptide-MHC docking, used high-resolution crystal structure of HLA-DRB1 complexed with Type II collagen peptide (PDB ID: 6BIN). back-end data for docking parameters is not available, and no independent validation of docking results	N/A No docking performed.	N/A No docking performed	N/A No docking performed
Detection Bias (Validation of Results)	High No experimental validation	High No experimental validation	Low Validated with ELISA and in vivo models	High No experimental validation
Reporting Bias (Transparency in Findings)	CD No negative results reported or study limitations.	CD No negative results reported or study limitations.	Moderate Limitations were discussed	Moderate Limitations were discussed
Overall appraisal	Include	Include	Include	Include

CD cannot determine.

## Data Availability

The original contributions presented in the study are included in the article. Further inquiries can be directed to the corresponding author.

## Supplementary Materials

The following supporting information can be downloaded at: https://www.mdpi.com/article/10.3390/pathogens15020152/s1, Supplementary S1: PECO elements; Supplement S2: Search term and search strategies; Supplement S3: JBI Critical Appraisal Checklist; Supplement S4: NIH Quality Assessment; Supplement S5: Risk of Bias Assessment Guidelines for computational studies; Supplement S6: List of excluded studies [57,58,59,60]; Supplement S7: List of all human sharing homology with viral peptides, their function and potential consequences of mimicry on immune response.

## Abbreviations

The following abbreviations are used in this manuscript:

CHIKV	Chikungunya virus
MAYV	Mayaro virus
SINV	Sindbis virus
SFV	Semliki forest virus
ONNV	O’nyong’nyong virus
RA	Rheumatoid arthritis
Anti-ccp	Anti-cyclic citrullinated peptide
ANA	Antinuclear antibody
C3 and 5	Complement 3 and 5
CII	Collagen II
MHC	Major histocompatibility complex
IL	Interleukin
