# Supplementary material for: Autoantibodies and Molecular Mimicry in Alphavirus Chronic Arthritis: A Systematic Review
Source: Pathogens. 2026 Jan 30;15(2):152. doi: 10.3390/pathogens15020152 (PMC12943495; doi:10.3390/pathogens15020152)
Supplement: Supplementary file 1 [file pathogens-15-00152-s001.zip › pathogens-4038107-supplementary.pdf]

Supplementary Material

Autoantibodies and molecular mimicry in alphavirus chronic arthritis: A systematic Review

NZ Masoto[1], FJ Burt[1,2*]

[1] Pathogen Research Laboratory, Division of Virology, Faculty of Health Sciences, University of the Free State, Bloemfontein, South Africa
[2] Division of Virology, National Health Laboratory Service (NHLS), Universitas, Faculty of Health Sciences, University of the Free State, Bloemfontein
* Correspondence: FJ Burt (BurtFJ@ufs.ac.za)

*** Correspondence:**
FJ Burt
BurtFJ@ufs.ac.za

Table of Contents

1 SUPPLEMENT S1 3

1.1 Systematic Review questions 3

1.2 PECO elements 3

1.2.1 PECO framework for question 1 3

1.2.2 PECO framework for question 2 3

1.3 PRISMA 3

2 SUPPLEMENT S2 8

2.1 SEARCH Strategy and SEARCH TERM 8

2.1.1 QUESTION 1 8

2.1.2 QUESTION 2 8

3 SUPPLEMENT S3 10

4 SUPPLEMENT S4 17

5 SUPPLEMENT S5 25

6 Supplement S6 29

1 SUPPLEMENT S1

1.1 Systematic Review questions

Question 1: Is there evidence of molecular or structural homology between alphavirus peptides and human proteins that may promote autoimmunity through molecular mimicry?

Question 2: Are autoantibodies associated with alphavirus-induced chronic arthritis, and do they have potential as predictive biomarkers for disease progression?

1.2 PECO elements

The multi-PECO elements were implemented for each question to ensure that the systematic review is sound.

1.2.1 PECO framework for question 1

I. Population – Arthritogenic Alphavirus

II. Exposure – Alphavirus peptides

III. Comparison – Human protein

IV. Outcome – Evidence of molecular mimicry (structural or molecular homology) in alphaviruses

1.2.2 PECO framework for question 2

i. Population – Patients with chronic arthritis after arthritogenic alphavirus infection

ii. Exposure – Autoantibodies

iii. Comparison – Acute patients, recovered patients or healthy controls.

iv. Outcome – Prevalence/presence of autoantibodies in chronic patients as potential biomarkers for autoimmunity

1.3 PRISMA

Section and Topic	Item #	Checklist item	Location where item is reported
**TITLE**			
Title	1	Identify the report as a systematic review.	Title of paper
**ABSTRACT**			
Abstract	2	See the PRISMA 2020 for Abstracts checklist.	Page 1
**INTRODUCTION**			
Rationale	3	Describe the rationale for the review in the context of existing knowledge.	Introduction page 2
Objectives	4	Provide an explicit statement of the objective(s) or question(s) the review addresses.	Introduction page 2 supplement material S1
**METHODS**			
Eligibility criteria	5	Specify the inclusion and exclusion criteria for the review and how studies were grouped for the syntheses.	Subsection "**The inclusion and exclusion criteria** " Page 4
Information sources	6	Specify all databases, registers, websites, organisations, reference lists and other sources searched or consulted to identify studies. Specify the date when each source was last searched or consulted.	Subsection "**The inclusion and exclusion criteria** " Page 4
Search strategy	7	Present the full search strategies for all databases, registers and websites, including any filters and limits used.	Supplementary Material S2
Selection process	8	Specify the methods used to decide whether a study met the inclusion criteria of the review, including how many reviewers screened each record and each report retrieved, whether they worked independently, and if applicable, details of automation tools used in the process.	Subsection "**Quality assessment and data extraction"** Page 5
Data collection process	9	Specify the methods used to collect data from reports, including how many reviewers collected data from each report, whether they worked independently, any processes for obtaining or confirming data from study investigators, and if applicable, details of automation tools used in the process.	Subsection "**Quality assessment and data extraction"** Page 5
Data items	10a	List and define all outcomes for which data were sought. Specify whether all results that were compatible with each outcome domain in each study were sought (e.g. for all measures, time points, analyses), and if not, the methods used to decide which results to collect.	No
	10b	List and define all other variables for which data were sought (e.g. participant and intervention characteristics, funding sources). Describe any assumptions made about any missing or unclear information.	No
Study risk of bias assessment	11	Specify the methods used to assess risk of bias in the included studies, including details of the tool(s) used, how many reviewers assessed each study and whether they worked independently, and if applicable, details of automation tools used in the process.	Subsection "**Quality assessment and data extraction"** Page 5. Supplementary material S3, S4 and S5**

Section and Topic	Item #	Checklist item	Location where item is reported
Effect measures	12	Specify for each outcome the effect measure(s) (e.g. risk ratio, mean difference) used in the synthesis or presentation of results.	No
Synthesis methods	13a	Describe the processes used to decide which studies were eligible for each synthesis (e.g. tabulating the study intervention characteristics and comparing against the planned groups for each synthesis (item #5)).	Subsection "**The inclusion and exclusion criteria** " Page 4
	13b	Describe any methods required to prepare the data for presentation or synthesis, such as handling of missing summary statistics, or data conversions.	No
	13c	Describe any methods used to tabulate or visually display results of individual studies and syntheses.	No
	13d	Describe any methods used to synthesize results and provide a rationale for the choice(s). If meta-analysis was performed, describe the model(s), method(s) to identify the presence and extent of statistical heterogeneity, and software package(s) used.	No
	13e	Describe any methods used to explore possible causes of heterogeneity among study results (e.g. subgroup analysis, meta-regression).	No
	13f	Describe any sensitivity analyses conducted to assess robustness of the synthesized results.	No
Reporting bias assessment	14	Describe any methods used to assess risk of bias due to missing results in a synthesis (arising from reporting biases).	No
**RESULTS**			
Study selection	16a	Describe the results of the search and selection process, from the number of records identified in the search to the number of studies included in the review, ideally using a flow diagram.	Subsection "Study selection" Page 6
	16b	Cite studies that might appear to meet the inclusion criteria, but which were excluded, and explain why they were excluded.	Supplement material S6
Study characteristics	17	Cite each included study and present its characteristics.	Subsection "Characteristics of included studies" Page 7-8, Table 2 and Table 3
Risk of bias in studies	18	Present assessments of risk of bias for each included study.	Subsection "Risk of bias assessment", Page 8-11, Table 4 and Table 5
Results of individual studies	19	For all outcomes, present, for each study: (a) summary statistics for each group (where appropriate) and (b) an effect estimate and its precision (e.g. confidence/credible interval), ideally using structured tables or plots.	Table 2

Section and Topic	Item #	Checklist item	Location where item is reported
Results of syntheses	20a	For each synthesis, briefly summarise the characteristics and risk of bias among contributing studies.	Table 2-5
	20b	Present results of all statistical syntheses conducted. If meta-analysis was done, present for each the summary estimate and its precision (e.g. confidence/credible interval) and measures of statistical heterogeneity. If comparing groups, describe the direction of the effect.	No
	20c	Present results of all investigations of possible causes of heterogeneity among study results.	Table 2-3
	20d	Present results of all sensitivity analyses conducted to assess the robustness of the synthesized results.	No
Reporting biases	21	Present assessments of risk of bias due to missing results (arising from reporting biases) for each synthesis assessed.	No
Certainty of evidence	22	Present assessments of certainty (or confidence) in the body of evidence for each outcome assessed.	No
**DISCUSSION**			
Discussion	23a	Provide a general interpretation of the results in the context of other evidence.	**Subsection "Evidence of Molecular Mimicry Between Alphavirus Peptides and Human Proteins"** page 12-13<br><br>**Subsection" Autoantibodies as Indirect Markers of Autoimmunity and Potential Predictive Biomarkers**<br>**of Chronicity"** page 13-14
	23b	Discuss any limitations of the evidence included in the review.	Conclusion, Page 16
	23c	Discuss any limitations of the review processes used.	**Subsection: "Limitations, Future Directions and Therapeutic Implications" page 15**
	23d	Discuss implications of the results for practice, policy, and future research.	Conclusion, Page 16

From: Page MJ, McKenzie JE, Bossuyt PM, Boutron I, Hoffmann TC, Mulrow CD, et al. The PRISMA 2020 statement: an updated guideline for reporting systematic reviews. BMJ 2021;372:n71. doi: 10.1136/bmj.n71

For more information, visit: http://www.prisma-statement.org/

Prisma 2020 for Abstracts Checklist

Section and Topic	Item #	Checklist item	Reported (Yes/No)
**TITLE**			
Title	1	Identify the report as a systematic review.	Yes
**BACKGROUND**			
Objectives	2	Provide an explicit statement of the main objective(s) or question(s) the review addresses.	Yes
**METHODS**			
Eligibility criteria	3	Specify the inclusion and exclusion criteria for the review.	Yes
Information sources	4	Specify the information sources (e.g. databases, registers) used to identify studies and the date when each was last searched.	Yes
Risk of bias	5	Specify the methods used to assess risk of bias in the included studies.	Yes
Synthesis of results	6	Specify the methods used to present and synthesise results.	Yes
**RESULTS**			
Included studies	7	Give the total number of included studies and participants and summarise relevant characteristics of studies.	Yes
Synthesis of results	8	Present results for main outcomes, preferably indicating the number of included studies and participants for each. If meta-analysis was done, report the summary estimate and confidence/credible interval. If comparing groups, indicate the direction of the effect (i.e. which group is favoured).	Yes
**DISCUSSION**			
Limitations of evidence	9	Provide a brief summary of the limitations of the evidence included in the review (e.g. study risk of bias, inconsistency and imprecision).	Yes
Interpretation	10	Provide a general interpretation of the results and important implications.	Yes
**OTHER**			
Funding	11	Specify the primary source of funding for the review.	No
Registration	12	Provide the register name and registration number.	No

From: Page MJ, McKenzie JE, Bossuyt PM, Boutron I, Hoffmann TC, Mulrow CD, et al. The PRISMA 2020 statement: an updated guideline for reporting systematic reviews. BMJ 2021;372:n71. doi: 10.1136/bmj.n71 For more information, visit: http://www.prisma-statement.org/

2 SUPPLEMENT S2

2.1 SEARCH Strategy and SEARCH TERM

2.1.1 QUESTION 1

Pubmed

("alphavir*"[All Fields] OR ("chikungunya fever"[MeSH Terms] OR ("chikungunya"[All Fields] AND "fever"[All Fields]) OR "chikungunya fever"[All Fields] OR "chikungunya"[All Fields]) OR "sindbis"[All Fields] OR ("ross"[All Fields] AND ("river s"[All Fields] OR "rivers"[MeSH Terms] OR "rivers"[All Fields] OR "river"[All Fields])) OR "mayaro"[All Fields] OR "o'nyong'nyong"[All Fields] OR "semliki"[All Fields]) AND ("molecular mimicry"[MeSH Terms] OR ("molecular"[All Fields] AND "mimicry"[All Fields]) OR "molecular mimicry"[All Fields] OR (("molecular"[All Fields] OR "moleculars"[All Fields]) AND ("homologies"[All Fields] OR "homology"[All Fields])) OR ("autoimmune"[All Fields] OR "autoimmunity"[MeSH Terms] OR "autoimmunity"[All Fields] OR "autoimmunities"[All Fields] OR "autoimmunization"[All Fields] OR "autoimmunizing"[All Fields]))

Scopus.

TITLE-ABS-KEY ((alphavir* OR chikungunya OR sindbis OR ross AND river OR mayaro OR o'nyong'nyong OR semliki) AND (molecular AND mimicry OR molecular AND homology OR autoimmunity))

ALL ((Alphavir* OR chikungunya OR sindbis OR "ross river" OR mayaro OR o'nyong'nyong OR Semliki) AND ("molecular mimicry" OR "molecular homology" OR autoimmunity))

Web of science

https://www.webofscience.com/wos/woscc/summary/c6c2733a-cdd9-471d-85e5-bda27fb690a9-01563f2e8f/relevance/1

ALL=((alphavir* or chikungunya or sindbis or ross river or mayaro or o'nyong'nyong or semliki) and (molecular mimicry or molecular homology or autoimmunity))

2.1.2 QUESTION 2

Pubmed

(("alphavir*"[All Fields] OR ("chikungunya fever"[MeSH Terms] OR ("chikungunya"[All Fields] AND "fever"[All Fields]) OR "chikungunya fever"[All Fields] OR "chikungunya"[All Fields]) OR "sindbis"[All Fields] OR ("ross"[All Fields] AND ("river s"[All Fields] OR "rivers"[MeSH Terms] OR "rivers"[All Fields] OR "river"[All Fields])) OR "mayaro"[All Fields] OR "o'nyong'nyong"[All Fields] OR "semliki"[All Fields]) AND ("autoantibodies"[MeSH Terms] OR "autoantibodies"[All Fields] OR "autoantibody"[All Fields] OR ("anti citrullinated protein antibodies"[MeSH Terms] OR ("anti citrullinated"[All Fields] AND "protein"[All Fields] AND "antibodies"[All Fields]) OR "anti citrullinated protein antibodies"[All Fields] OR ("anti"[All Fields] AND "ccp"[All Fields]) OR "anti ccp"[All Fields]) OR ("rf"[Journal] OR "rf"[All Fields]) OR "ANA"[All Fields] OR ("autoimmune"[All Fields] OR "autoimmunity"[MeSH Terms] OR "autoimmunity"[All Fields] OR "autoimmunities"[All Fields] OR "autoimmunization"[All Fields] OR "autoimmunizing"[All Fields]))) AND (("chronic"[All Fields] OR "chronical"[All Fields] OR "chronically"[All Fields] OR "chronicities"[All Fields] OR "chronicity"[All Fields] OR "chronicization"[All Fields] OR "chronics"[All Fields]) AND ("arthritis"[MeSH Terms] OR "arthritis"[All Fields] OR "arthritides"[All Fields] OR "polyarthritides"[All Fields]))

("alphavir*"[All Fields] OR ("chikungunya fever"[MeSH Terms] OR ("chikungunya"[All Fields] AND "fever"[All Fields]) OR "chikungunya fever"[All Fields] OR "chikungunya"[All Fields]) OR "sindbis"[All Fields] OR ("ross"[All Fields] AND ("river s"[All Fields] OR "rivers"[MeSH Terms] OR "rivers"[All Fields] OR "river"[All Fields])) OR "mayaro"[All Fields] OR "o'nyong'nyong"[All Fields] OR "semliki"[All Fields]) AND ("autoantibodies"[MeSH Terms] OR "autoantibodies"[All Fields] OR "autoantibody"[All Fields] OR ("anti citrullinated protein antibodies"[MeSH Terms] OR ("anti citrullinated"[All Fields] AND "protein"[All Fields] AND "antibodies"[All Fields]) OR "anti citrullinated protein antibodies"[All Fields] OR ("anti"[All Fields] AND "ccp"[All Fields]) OR "anti ccp"[All Fields]) OR ("rf"[Journal] OR "rf"[All Fields]) OR ("rheumatoid factor"[MeSH Terms] OR ("rheumatoid"[All Fields] AND "factor"[All Fields]) OR "rheumatoid factor"[All Fields]) OR "ANA"[All Fields] OR ("antinuclear"[All Fields] AND "antibod*"[All Fields]) OR ("autoimmune"[All Fields] OR "autoimmunity"[MeSH Terms] OR "autoimmunity"[All Fields] OR "autoimmunities"[All Fields] OR "autoimmunization"[All Fields] OR "autoimmunizing"[All Fields])) AND ((("chronic"[All Fields] OR "chronical"[All Fields] OR "chronically"[All Fields] OR "chronicities"[All Fields] OR "chronicity"[All Fields] OR "chronicization"[All Fields] OR "chronics"[All Fields]) AND ("arthritis"[MeSH Terms] OR "arthritis"[All Fields] OR "arthritides"[All Fields] OR "polyarthritides"[All Fields])) OR "musculoskeletal*"[All Fields])

Scopus

TITLE-ABS-KEY ((alphavir* OR chikungunya OR sindbis OR ross AND river OR mayaro OR o'nyong'nyong OR semliki) AND (autoantibodies OR anti-ccp OR rf OR rheumatoid AND factor OR ana OR antinuclear AND antibod* OR autoimmunity) AND (chronic AND arthritis OR musculoskeletal*))

ALL ((alphavir* OR chikungunya OR sindbis OR "ross river" OR mayaro OR o'nyong'nyong OR semliki) AND (autoantibodies OR anti-ccp OR rf OR "rheumatoid factor" OR ana OR "antinuclear antibod*" OR autoimmunity) AND (chronic AND arthritis OR musculoskeletal*))

Web of science

https://www.webofscience.com/wos/woscc/summary/67f4d6e6-8655-4605-ad80-56802b4ed5f0-01563f9734/relevance/1

ALL=((alphavir* or chikungunya or sindbis or ross river or mayaro or o'nyong'nyong or semliki) and (autoantibodies or anti-CCP or RF or rheumatoid factor or ANA or antinuclear antibod* or autoimmunity) and (chronic arthritis or musculoskeletal*))

3 SUPPLEMENT S3

JBI Critical Appraisal Checklist for analytical cross-sectional studies

JBI Criteria	Bashir et al., 2020 [28]	Chang et al., 2018 [17]	Sengupta et al., 2023 [29]	Chopra and Venugopalan 2011 [30]
Were the criteria for inclusion in the sample clearly defined?	✓ Yes<br><br>Patients with clinically and serologically confirmed CHIKV infection. The study excluded individuals with known rheumatic diseases (e.g., RA, SLE, Sjögren's), sarcoidosis, chronic infection, and cancer.	✓ Yes<br><br>Participants were adults ≥18 years with clinically or laboratory-confirmed CHIKV infection and chronic arthritis	✓ Yes<br><br>Participants had to present acute febrile illness with chikungunya-like symptoms and confirmed CHIKV infection. Exclusions included patients with hematologic malignancies, liver/kidney disease, and other confounding illnesses	✓ Yes<br><br>Villagers with persistent aches and pains and a past history of acute Chikungunya illness.
Were the study subjects and the setting described in detail?	✓ Yes<br><br>The setting (Port Sudan private medical center) and timeframe (March–September 2019) were clearly stated. Demographics are provided.	✓ Yes<br><br>Subjects were well described, including demographics The setting was clearly defined as the Atlántico and Bolívar Departments in Colombia during the 2014–2015 epidemic.	✓ Yes<br><br>Participants were from the Calcutta School of Tropical Medicine, India, recruited between Sept 2014–Oct 2016. Demographics were well-documented	✓ Yes<br><br>The study subjects were villagers from Modnimb village, district Sholapur. The setting was a rural community-based study in village Modnimb, India,
Was the exposure measured in a valid and reliable way?	✓ Yes<br><br>CHIKV infection was confirmed using ELISA	✓ Yes	✓ Yes<br><br>ELISA kits and real-time qRT-PCR methods. Blood	✓ Yes<br><br>A combination of self-reported history and serological

		Confirmed using validated ELISA tests for IgG/IgM	samples were tested in triplicate	testing for anti-CHIKV IgG and IgM antibodies
Were objective, standard criteria used for measurement of the condition?	✓ Yes<br><br>Joint conditions (arthritis, arthralgia) were classified based on the ACR disease activity score	✓ Yes<br><br>Chronic arthritis was assessed using established tools like the DAS-28 (Disease Activity Score-28)	✓ Yes<br><br>Arthritis was assessed using the 2010 ACR/EULAR classification criteria	✓ Yes<br><br>Standardized case record forms, serological tests, and established diagnostic criteria to measure the condition
Were confounding factors identified?	✓ Yes<br><br>Sex and comorbidities	✓ Yes<br><br>age, gender and education level	✓ Yes<br><br>Age, sex, genotype	✓ Yes<br><br>Age, sex
Were strategies to deal with confounding factors stated?	✓ Yes<br><br>The Kolmogorov-Smirnov test was employed to assess the distributions of continuous variables and it exhibited that the variables were normally distributed for each group. participants with comorbidities were excluded, At the point when patients were arranged by sex, it was noted that both genders were equally prone to have CHIKV infection symptoms	Yes,<br><br>Participants were comparable in terms of age, gender, ethnicity, and education level<br><br>chi-square or Fishers Exact test were used to compare categorical variables and analysis of variance for normally distributed continuous variables	✓ Yes<br><br>Multivariate analyses (e.g., ANOVA), and ROC curve analysis to assess diagnostic performance and reduce confounding. Cases and controls were age and ethnicity matched	No
Were the outcomes measured in a valid and reliable way?	✓ Yes<br><br>Measured using validated laboratory	✓ Yes<br><br>qRT-PCR, viral culture, ELISA, mass spectrometry, and	✓ Yes	✓ Yes<br><br>The study used standardized clinical

	tests and the ACR arthritis activity score	standardized clinical scoring tools.	Biomarkers were measured using commercial ELISA	evaluation, serological tests
Was appropriate statistical analysis used?	✓ Yes<br><br>Mann-Whitney, Wilcoxon, Chi-square, Fisher's exact, Spearman correlation, and regression analysis.	✓ Yes<br><br>square, Fisher's exact, t-tests, ANOVA, and Kruskal-Wallis for non-parametric data	✓ Yes<br><br>Chi-square tests, t-tests, ANOVA, ROC curve analysis, correlation analysis, and principal component analysis (PCA). P-values, ORs, confidence intervals, and additive genetic models were properly reported	✓ Yes<br><br>SPSS statistical software package statistical analysis; level of significant P < 0.05. Geometric mean was computed for sera-cytokine data. Significant differences between groups were tested by ANOVA
Overall Appraisal (Include / Exclude / Seek Further Information)	Include	Include	Include	Include
Comments / Reason for Exclusion	Recalling bias may have occurred because the study relied on participants' retrospective self-reporting of symptoms such as joint pain, its duration, and severity, some of which occurred months before data collection			

JBI Critical Appraisal Checklist for cohort studies

JBI Checklist Questions	Schilte et al. (2013) [33]	Guillot et al. (2020)* [31]	Manimunda et al. (2010)[32]

1. Were the two groups similar and recruited from the same population?	Yes<br><br>viremic patients from the same outbreak and hospital	Yes<br><br>Patients with confirm chikv, recruited from the same medical center in Reunion Island.	Yes<br><br>Patients were recruited from primary health centers
2. Were the exposures measured similarly to assign people to both exposed and unexposed groups?	Yes<br><br>Exposure was confirmed using RT-PCR	Yes<br><br>Exposure was confirmed via serology retrospectively.	Yes<br><br>Exposure confirmed via IgM serology
3. Was the exposure measured in a valid and reliable way?	Yes<br><br>RT-PCR a Gold standard	Yes<br><br>ELISA is a reliable diagnostic tool	Yes
4. Were confounding factors identified?	No	No	Yes<br><br>age
5. Were strategies to deal with confounding factors stated?	No	No	Yes<br><br>Confounders were adjusted by stratifying sampling of participants according to age groups
6. Were the groups/participants free of the outcome at the start of the study (or at the moment of exposure)?	Yes<br><br>All participants were in the acute phase of CHIKV infection at baseline	No<br><br>had musculoskeletal pain at the beginning of follow-up	Yes<br><br>Participants were in the acute phase of infection
7. Were the outcomes measured in a valid and reliable way?	Yes<br><br>Clinical assessments and serological markers were used, lab testing.	Yes<br><br>Joint symptoms were assessed through clinical evaluations and imaging	Yes<br><br>Xray and MRI. anti-CCP antibody was tested by ELISA, RF was tested by nephelometry
8. Was the follow-up time reported and sufficient to be	Yes	Yes<br><br>13-year follow-up period	Yes<br><br>Follow-up was 10 months

long enough for outcomes to occur?	Follow-up was conducted for 36 months		
9. Was follow-up complete, and if not, were the reasons for loss to follow-up described and explored?	No<br><br>Some loss to follow-up, reasons not always detailed	No<br><br>High loss to follow-up (58% loss).	Yes<br><br>The follow-up was well maintained
10. Were strategies to address incomplete follow-up utilized?	No	No	N/A
11. Was appropriate statistical analysis used?	Yes<br><br>Logistic regression was used	Yes<br><br>non-parametric Wilcoxon Mann-Whitney tests for quantitative<br><br>values and Fisher tests for qualitative values.	Yes<br><br>Prevalence and subgroup analysis were conducted
Overall Appraisal	Include	Include	Include
Comments (Including reason for exclusion)	lacks confounder adjustment	The study compares two groups of participants with chronic arthritis. The group that recovered and the group that continued to have chronic arthritis even after 13 years of follow up, hence why participants were not free of outcome at the beginning of the study	

JBI Checklist for Case Series

JBI Criteria	Sepúlveda Delgado et al,2017 [35]	Chopra et al 2008 [36]
1. Clear inclusion criteria?	✅ Yes	✅ Yes

	consecutive patients with ChikV infection from January to April 2015.	patients diagnosed with chikungunya fever during an outbreak and tracks their musculoskeletal symptoms.
2. Was the condition measured in a standard, reliable way for all participants?	✅ Yes<br><br>Standardized methods (DAS-28, WHODAS-II, biomarkers).	✅ Yes<br><br>Chikungunya fever was diagnosed based on clinical criteria
3. Were valid methods used for the identification of the condition for all participants included in the case series	✅ Yes<br><br>ChikV infection was confirmed by RT-PCR and for evaluation of severity, DAS-28 index was used	✅ Yes<br><br>The study relies on physician diagnosis and ELISA
4. Did the case series have consecutive inclusion of participants?	✅ Yes<br><br>Patients were included consecutively from January to April 2015	❓ Unclear
5. Did the case series have complete inclusion of participants	❓ Unclear<br><br>No details on dropouts except for one patient who died and it's unclear if those were all the patient with the infection at that specific location at the time	❓ Unclear<br><br>There is no mention of whether all cases from the outbreak were considered or if some were excluded.
6.Was there clear reporting of the demographics of the participants in the case series?	Age, gender, comorbidities, and joint involvement reported.	❓ unclear<br><br>Only gender was reported, more information is needed on age and comorbidities
7.Was there clear reporting of clinical information of the participants?	✅ Yes<br><br>Symptoms, joint involvement, and evolution described.	Yes<br><br>symptoms, disease course, and musculoskeletal manifestations are described in detail.
8. Were the outcomes or follow-up results of cases clearly reported?	✅ Yes	❓ Unclear<br><br>The study tracks symptoms over time but does not provide structured follow-up

	Follow-up data provided for 12 months.	intervals or describe how many participants were lost to follow-up
9.Was there clear reporting of the presenting site(s)/clinic(s) demographic information?	✅ Yes<br><br>Study conducted in a tertiary care hospital in Mexico.	NO<br><br>The study does not specify hospital locations or recruitment centers.
10.Were statistical methods appropriate?	✅ Yes<br><br>Wilcoxon rank-sum and Spearman correlation used.	No<br><br>Only descriptive statistics were used (e.g., frequencies, percentages), with no advanced statistical techniques to analyze trends over time
JBI Risk of Bias Judgment: LOW RISK	Include	High risk of Bias<br><br>The study has several methodological weaknesses, including unclear participant selection, lack of control over follow-up, and minimal statistical analysis.

JBI Critical Appraisal Checklist for Case Reports: (De Carvalho et,al, 2021) [57]

JBI Criteria	Assessment	Comments
1. Were patient's demographic characteristics clearly described?	✅ Yes	Age, gender, and medical background were detailed.
2. Was the patient's history clearly described?	✅ Yes	The clinical timeline was included.
3. Were the clinical condition and diagnostic tests clearly described?	✅ Yes	CHIKV serology, ANA, and autoantibody testing were included.
4. Was the intervention(s) or treatment clearly described?	✅ Yes	Hydroxychloroquine, methotrexate, and betamethasone were mentioned.
5. Was the post-intervention condition clearly described?	✅ Yes	The response to treatment was stated.
6. Were adverse events reported?	❌ No	The study lacks explicit discussion of potential adverse effects.

7. Does the case report provide takeaway lessons?	✅ Yes	Highlights molecular mimicry in CHIKV-associated autoimmunity.
**Overall Risk of Bias**	**Low Risk**	The study is well-documented but lacks discussion on adverse events.

4 SUPPLEMENT S4

NIH Quality Assessment Tool for Observational Cohort and Cross-Sectional Studies

**NIH Quality Assessme nt**	**Schilte et al. (2013) [33]**	**Guillot et al. (2020) [31]**	**Manimun da et al. (2010) [32]**	**Bashir & El-Dirdiri (2020) [28]**	**Chang et al. (2018) [17]**	**Sengupta et al. (2023) [29]**	**Chopra and Venugopalan [30]**
**Was the research question or objective clearly stated?**	✅ Yes<br>The study aimed to evaluate the conseque nces of long-term arthralgia on patients' daily and social life, looked for risk factors associated with them and estimated their economic impact	✅ Yes<br>The study aimed to assess long-term joint symptoms following Chikungunya infection	✅ Yes<br>Yes – The study assessed disease progressio n post-Chikungu nya.	✅ Yes<br>The study aimed to assess post-Chikungu nya joint pain sequelae in Sudan.	✅ Yes<br>The study aimed to assess the persistenc e of CHIKV in synovial fluid.	✅ Yes<br>The study assessed biomarker s for post-Chikungu nya chronic arthritis prognosis.	✅ Yes<br>Investigate the prevalence and characteristics of persistent rheumatic musculoskeletal pain and disorders (RMSK) following a Chikungunya epi demic
**Was the study population clearly specified and defined?**	✅ Yes<br>A cohort of patients (n = 180) enrolled for febrile arthralgia to the	✅ Yes<br>serologically confirmed Chikungunya patients suffering from chronic musculoskelet al pain were	✅ Yes<br>The present study was carried out from June 2008<br>to April 2009 in	✅ Yes<br>Participan ts were CHIKV-confirmed patients from a single Sudanese	✅ Yes<br>Patients were recruited from the Atlantico and Bolívar Departme	✅ Yes<br>CHIKV-confirmed patients all age groups and sexes, during their first visit at	✅ Yes<br>villagers from two villages in rural southwestern Madagascar who had a history of acute Chikungunya virus (CHIKV)

	Emergency Department of the Groupe Hospitalier Sud Re´union between March 2005 and May 200	initially referred to a single rheumatologist (A.R.) in Saint-Denis 1741 teaching hospital (median time interval after the initial viral infection in 2005–2006: 38.5 months).	one of the panchayats of Dakshina Kannada District and based at a primary health centre	medical center	nts of Colombia.	Calcutta School of Tropical Medicine (CSTM), West Bengal, India from September 2014 to October 2016	illness during the 2006 epidemic.
**Was the participation rate of eligible persons at least 50%?**	✅ Yes Majority of eligible patients participated.	❌ No High loss to follow-up was reported, reducing representativeness.	✅ Yes 85.7% of eligible participants participated	✅ Yes all patients participated	✅ Yes Majority of eligible patients participated	✅ Yes Majority of eligible patients participated.	✅ Yes Majority of eligible patients participated.
**Were all subjects recruited from the same population and uniform eligibility criteria/ inclusion/exclusion criteria pre-established and applied uniformly**	✅ Yes All recruited from the same outbreak and hospital	✅ Yes All patients were recruited from the same medical center in Reunion Island.	✅ Yes Patients from primary health centers.	✅ Yes Patients with pre-existing rheumatologic diseases were excluded	✅ Yes Subjects were excluded if they reported a known bleeding disorder or were receiving anticoagulant medications. The study also excluded children, adults unable to give consent,	✅ Yes Patients with acute febrile illness and history of headache, body ache, myalgia, arthralgia, rash, with or without haemorrhagic manifestation of all age-groups	✅ Yes eligibility criteria were pre-established and applied uniformly, with participants being individuals who had a history of acute Chikungunya virus (CHIKV) illness during the 2006 epidemic.

					prisoners, and pregnant women	and both sexes were included.	
**Was a sample size justification or power analysis provided?**	❌ No<br><br>(NFL). This is not a fatal flaw according to guidelines since it's normal for observational studies not to report on such	❌ No<br><br>(NFL)- This is not a fatal flaw according to guidelines since it's normal for observational studies not to report on such	❌ No<br><br>(NFL)- This is not a fatal flaw according to guidelines since it's normal for observational studies not to report on such	✅ Yes<br><br>The gauging of sample size was calculated using a cross-sectional statistic formula at 95% CI, 18% marginal errors with 50% of CHIKV distributed in a similar study.	✅ Yes<br><br>The text provides sample size justification, power description, and variance and effect estimates. Power calculations are discussed, including alpha levels, effect sizes, and power values.	✅ Yes<br><br>population size was calculated using EpiInfo™ version 7.2 software of CDC, with 95% confidence interval	❌ No<br><br>(NFL)- This is not a fatal flaw according to guidelines since it's normal for observational studies not to report on such
**Was exposure assessed prior to outcome measurement?**	✅ Yes<br><br>Exposure was confirmed via qRT-PCR.	✅ Yes<br><br>Exposure to Chikungunya and the outcome was determined retrospectively.	✅ Yes<br><br>Exposure was confirmed before follow-up, Participants were Chikungunya virus IgM positive.	✅ Yes<br><br>CHIKV infection was confirmed before assessing joint symptoms	✅ Yes<br><br>CHIKV infection was confirmed before joint analysis.	✅ Yes<br><br>infection was confirmed before tracking arthritis development.	✅ Yes<br><br>CHIKV infection was confirmed before.
**Was the timeframe sufficient to observe an association?**	✅ Yes<br><br>36-month follow-up was sufficient.	✅ Yes<br><br>The study followed patients for 13 years,	✅ Yes<br><br>10-month follow-up was appropriate.	✅ Yes<br><br>Six months follow up	✅ Yes<br><br>22 months follow up	✅ Yes<br><br>Two-year follow-up was adequate.	✅ Yes<br><br>One-year follow-up

**Were different levels of exposure examined?**	N/A<br><br>The exposure is a dichotomous variable.	N/A<br><br>The exposure is a dichotomous variable.	N/A<br><br>The exposure is a dichotomous variable.	N/A<br><br>The exposure is a dichotomous variable.	N/A<br><br>The exposure is a dichotomous variable.	N/A<br><br>The exposure is a dichotomous variable.	N/A<br><br>The exposure is a dichotomous variable.
**Were the exposure measures clearly defined, valid, and reliable?**	✅ Yes<br><br>RT-PCR was used for confirmation.	✅ Yes<br><br>Exposure was confirmed using serology.	✅ Yes<br><br>IgM serology confirmed exposure.	✅ Yes<br><br>Chikv was confirmed by ELISA	✅ Yes<br><br>confirmation was done via serology and RT-PCR	✅ Yes<br><br>confirmed via serology and PCR.	✅ Yes<br><br>confirmed via indirect IFA
**Was the exposure assessed more than once?**	❌ No<br><br>Only initial confirmation	☑ Yes<br><br>The study reports the presence of virus IgG, 13 years post infection. Which means the exposure was assessed more than once	❌ No<br><br>Single measurement	❌ No<br><br>Only baseline measurement	❌ No<br><br>Single measurement	✅ Yes<br><br>✅ Yes – Follow-up data was collected over time	❌ No<br><br>Single measurement
**Were the outcome measures clearly defined, valid, and reliable?**	✅ Yes<br><br>Clinical assessments were conducted and laboratory testing.	✅ Yes<br><br>Yes – Joint symptoms were assessed through clinical evaluations and imaging	✅ Yes<br><br>X-ray and MRI. anti-CCP antibody was tested by ELISA, RF was tested by nephelometry	✅ Yes<br><br>Arthritis severity was measured using a validated scoring system.	✅ Yes<br><br>Yes – qRT-PCR for chikungunya virus RNA, mass spectrometry analysis for viral proteins. Disease Activity Score in 28 joints (DAS28) for	✅ Yes<br><br>Biomarkers were measured using validated ELISA.	✅ Yes<br><br>clinical examination by a rheumatologist

					arthritis severity		
**Were outcome assessors blinded to exposure status?**	NR<br><br>Blinding not reported.	❌ No<br><br>The person assessing the outcome is the main investigator in this study<br><br>which means the probability of the investigator knowing about exposure are high	NR<br><br>No mention of blinding.	NR<br><br>No mention of blinding.	NR<br><br>No mention of blinding.	NR<br><br>No mention of blinding.	NR<br><br>No mention of blinding
**Was loss to follow-up after baseline 20% or less?**	CD<br><br>Some loss to follow-up were temporarily, loss to follow up was not clearly reported	❌ No<br><br>High loss to follow-up (58% loss).	✅ Yes	✅ Yes<br><br>Follow-up was well maintained	✅ Yes<br><br>Follow-up was well maintained	✅ Yes<br><br>Follow-up was well maintained.	✅ Yes
**Were key potential confounding variables measured and adjusted for?**	N/A<br><br>Confounders such as age and gender are the factors of interest to the out objective of the research	❌ No<br><br>The study did not control for confounders like pre-existing conditions.	✅ Yes<br><br>Confounders were adjusted by stratifying sampling of participants according to age groups	✅ Yes<br><br>Participants with comorbidities were excluded, At the point when patients were arranged by sex, it was noted	✅ Yes<br><br>Key potential confounding variables (age, sex, ethnicity, and education level) were measured and found to be	✅ Yes<br><br>Yes – Statistical adjustments were made for genotype and biomarker levels. Cases and controls were age and	❌ No<br><br>The study did not control or measure for confounders

				that both genders were equally prone to have CHIKV infection symptoms .	comparabl e across groups, suggesting adjustmen t for these varia bles	gender matched	
**Overall Appraisal**	**Include**	**Include**	**Include**	**Include**	**Include**	**Include**	**Include**

NIH Assessment Tool for Before-After (Pre-Post) Studies with No Control Group ***Chopra et al. (2012)***

**Question**	**Chopra et al. (2012) [34]**	**Comments**
**Was the study question clearly stated?**	✅ Yes	**The study aimed to document the natural history of musculoskeletal symptoms following Chikungunya.**
**Were eligibility/selection criteria for participants prespecified and clearly described?**	✅ Yes	Inclusion criteria Residents of the study area, Aged 15 years or older Presence of acute Chikungunya infection (confirmed by IgM ELISA)
**Were participants representative of the population?**	✅ Yes	The study recruited a broad population from an affected rural area.
**Were all eligible participants included?**	✅ Yes	The study included all eligible community members.
**Was sample size sufficiently large to provide confidence in findings?**	✅ Yes	The sample size of 509 people with virus infection was sufficient due to the relatively high prevalence of RMSK sequelae (40%). Additionally, the study's objective of recording RMSK sequelae

		required less statistical power compared to studies examining associations or differences between groups
**Was the intervention (exposure) clearly described?**	✅ Yes	Chikungunya infection was described as the exposure of interest.
**Were outcome measures pre-specified, valid, and reliable?**	✅ Yes	Clinical assessments were conducted following a structured protocol and laboratory methods were used.
**Were outcome assessors blinded to exposure status?**	NR	Blinding was not reported
**Was follow-up sufficiently long to detect an effect?**	✅ Yes	The study followed participants for two years, allowing assessment of long-term symptoms
**Was loss to follow-up ≤20%?**	**CD**	Only patients who continue to show symptoms were followed up, all recovered patients were dropped in each follow up period
**Were statistical tests appropriate for before-after comparisons?**	✅ Yes	Statistical analyses were performed to track changes over time.
**Were multiple outcome measures assessed before and after the exposure?**	✅ Yes	Symptoms and inflammatory markers were evaluated at multiple time points
**Was there any attempt to minimize confounding variables?**	❌ No	No statistical adjustments for confounders were made.
**Were missing data handled appropriately?**	✅ Yes	Missing data were accounted for in the analysis.
**Overall Appraisal**		Include

NIH Assessment Tool for case series (Sepúlveda-Delgado et al)

NIH Criteria	Sepúlveda-Delgado et al 2017 [35]	Chopra et al, 2008 [36]
1. Clearly stated research question?	✅ Yes<br><br>Case series to evaluate the relationship of IL-6 and other	✅ Yes<br><br>The study aims to assess the clinical progression and long-term

	inflammatory biomarkers with the severity and clinical evolution of CAD after Chikungunya fever	musculoskeletal symptoms of chikungunya fever.
2. Clearly described study population?	✅ Yes<br><br>A prospective case series was performed in a tertiary care center during the Mexican ChikV outbreak. It included consecutive patients with ChikV infection from January to April 2015	✅ Yes<br><br>patients with post-CHIK musculoskeletal disorders
3. Were the cases consecutive	✅ Yes<br><br>Patients were included sequentially infection from January to April 2015	NR<br><br>It is unclear whether all patients from the outbreak were included
4. Did the authors include patients at the same stage of disease	✅ Yes<br><br>All had acute ChikV infection.	✅ Yes<br><br>all patients had acute infection and musculoskeletal disorders
5. All eligible participants included?	Unclear if any patients were excluded beyond the reported death.	✅ Yes<br><br>All eligible patients were included
6. Was the intervention (if any) clearly described	N/A<br><br>No intervention was applied; only observational.	N/A<br><br>No intervention was applied; only observational.
7.Were the outcomes clearly defined, valid, reliable, and measured consistently across all study participants?	✅ Yes<br><br>DAS-28, WHODAS-II, and biomarkers measured at multiple time points.	CD – the outcomes were defined however method of measurements were not reported, cannot determine reliability.
8.Was the length of follow-up adequate	✅ Yes<br><br>12-month follow-up conducted.	CD<br><br>The study mentions **follow-up** but does not specify duration for all patients.
9. Were the statistical methods well-described	✅ Yes	NO

	Wilcoxon test and Spearman correlation coefficient	The study only reports **descriptive statistics** (percentages, frequencies) and does not use **inferential statistics** to assess relationships
10.Was there an appropriate reporting of results	✅ Yes<br><br>Findings were clearly reported in tables and text	✅ Yes<br><br>The study provides a clear breakdown of symptoms and progression over time.
NIH Risk of Bias Judgment: LOW RISK	Include	High risk of bias. The study has several methodological weaknesses, including unclear participant selection, lack of control over follow-up, and minimal statistical analysis.

5 SUPPLEMENT S5

Risk of Bias Assessment Guidelines for computational studies.

1. **Selection Bias**

What It Means:

Selection bias occurs when the data sources (e.g., protein sequences, molecular structures) are not representative of the full range of possible options, leading to biased results.

What Should Be Considered:

- ✅ Ensure a broad and unbiased selection of biological entities (e.g., viral strains, bacterial species, host proteins, genomes, viral proteome) relevant to the study.
- ✅ Clearly define criteria for selecting sequences or structural data.
- ❌ Avoid cherry-picking data that supports a hypothesis while ignoring contradictory evidence.
- ❌ Avoid focusing on subsets of protein without explaining why others were excluded

How to Assess It:

- Low Risk: Data sources are comprehensive, covering diverse and relevant biological and computational variables.
- Moderate Risk: Some limitations in dataset selection, but the study includes diverse, relevant inputs.
- High Risk: Narrow focus on a specific dataset or biological entity without justification, limiting generalizability.

2. **Input Data Preparation & Optimization**

What It Means

Proper preparation of protein sequences, molecular structures, and ligand data is crucial for accurate computational analysis. Poorly processed data can lead to misleading predictions in bioinformatics, molecular docking, and structural modeling.

What Should Be Considered:

- ✅ Use well-established databases (e.g., UniProt, GenBank, PDB) for selecting proteins, peptides, and ligands
- ✅ Use appropriate software for input preparation and alignment optimization (e.g., BLAST, Clustal Omega, or Peptide Match) (e.g., Open Babel, LigPrep for ligand optimization; GROMACS for molecular dynamics, fastaQC for read quality, BLAST, Clustal Omega, or Peptide Match).
- ✅ Ensure physiological relevance of data (e.g., pH adjustments, ionization states, biological temperature).
- ✅ Apply quality checks on input data (e.g., RMSD for docking, energy minimization for structural models).
- and curation of protein sequence (e.g. remove duplicates, coring errors)
- ❌ Avoid using unprocessed or raw input data without proper optimization.

How to Assess It:

- Low Risk: Inputs are thoroughly optimized and biologically/chemically accurate.
- Moderate Risk: Some optimization performed, but not all conditions considered.
- High Risk: No optimization or quality checks performed, leading to unreliable results.

---

3. Performance Bias (Computational Methods & Software Use)

What It Means:

Performance bias arises when the computational methods (e.g., sequence alignment, molecular docking) are not properly chosen or standardized, leading to unreliable predictions.

What Should Be Considered:

- ✅ Use validated bioinformatics and computational tools (e.g., MUSCLE for sequence alignment, AutoDock for docking, IEDB for epitope prediction).
- ✅ Standardize parameters across different tools to ensure consistency.
- ✅ Compare results with alternative computational methods to reduce bias (Cross-validation with other methods)
- ❌ Do not rely on a single method without justification, as different tools may yield different results.
- ❌ Avoid using outdated or low-accuracy software when better alternatives exist.

How to Assess It:

- Low Risk: Used validated computational tools with clear standardization and cross-validation.
- Moderate Risk: Used appropriate tools, but some parameters were inconsistent.
- High Risk: No validation or standardization, leading to potentially unreliable results.

---

4. Target Selection (For Molecular Docking Studies)

What It Means:

The protein structure (target) used in molecular docking must be high-quality and biologically relevant.

What Should Be Considered:

- ✅ Use protein structures with high resolution (≤2.5 Å from X-ray crystallography or cryo-EM).
- ✅ Ensure the protein structure matches physiological conditions (e.g., correct histidine protonation).
- ✅ Validate computational models (e.g., homology models) against experimental data if no empirical structures are available.
- ❌ Avoid using low-resolution structures (>2.5 Å), as they may introduce errors.
- ❌ Avoid using homology models without validation, as they may not be reliable.

How to Assess It:

- Low Risk: High-resolution, experimentally determined structure used.
- Moderate Risk: Used homology modeling but validated against known structures.
- High Risk: Used low-quality or unverified protein structures.

---

5. Detection Bias (Validation of Results)

What It Means:

Detection bias occurs when *in silico* results are not validated, making it unclear whether they reflect real biological processes. Were outcomes validated or cross-checked?

What Should Be Considered:

- ✅ Compare results with experimental data (e.g., ELISA, crystallography, immune assays, in vitro/in vivo models).
- ✅ Use independent datasets to confirm findings.
- ✅ Validate docking results with re-docking (RMSD check).
- ❌ Do not rely entirely on computational predictions without validation.

How to Assess It:

- Low Risk: Validated with experimental data and independent datasets.
- Moderate Risk: Some validation was done, but key findings remain computational.
- High Risk: No validation—findings are purely theoretical.

---

6. Reporting Bias (Transparency in Findings)

What It Means: Reporting bias arises when only favorable results are presented, leading to a skewed interpretation.

What Should Be Considered:

- ✅ Report both positive and negative findings
- ✅ Document any alternative explanations for unexpected results.

- ✅ Clearly state study limitations and potential biases.

How to Assess It:

- Low Risk: Reports all findings, including negative or unexpected results.
- Moderate Risk: Some reporting bias, but alternative explanations are given.
- High Risk: Only positive results are reported negative findings omitted.

---

7. External Validity (Reproducibility)

What It Means:

External validity assesses whether computational findings can be reproduced by others.

What Should Be Considered:

- ✅ Ensure computational methods are reproducible by providing all parameters and data sources.
- ❌ Avoid non-reproducible methods (e.g., proprietary software without disclosure of parameters).

How to Assess It:

- Low Risk: Findings are reproducible
- Moderate Risk: Findings are partially reproducible
- High Risk: Findings cannot be reproduced independently.

6 SUPPLEMENT S6

List of excluded studies that meets the inclusion criteria

Studies	Reason for exclusion
(Amaral & Schoen, 2018) [51]	A case report
(Chopra et al., 2008) [36]	High risk of bias
(de Carvalho et al., 2021) [57]	A case report
(Imai et al., 2016) [58]	A case report
(Maek-a-nantawat & Silachamroon, 2009) [59]	A case report
(Sane et al., 2012) [60]	Unclear reporting of which group were autoantibodies detected

7 SUPPLEMENT S7

List of all human sharing homology with viral peptides, their function and potential consequences of mimicry on immune response

**Venigalla et.al., 2020 -A possible role for autoimmunity through molecular mimicry in alphavirus mediated arthritis**			
**Viral peptide**	**Human Protein**	**Normal Function (UniProt)**	**Potential Viral Mimicry Impact on Immune Response**
FIPTQTFY	**Ryanodine receptor 1 (RyR1)**	Skeletal muscle calcium channel for contraction.	Cross-reactive antibodies trigger autoimmune myopathy and muscle weakness.
KGRVVAIVLGGANEGARTALSVVTW	**Interleukin-17 receptor C (IL-17RC)**	Mediates IL-17 inflammatory signaling.	Dysregulated IL-17 signaling drives chronic inflammation and autoimmunity.
TSAPCTITGTMGHFILARCPKG	**CD14**	Co-receptor for LPS detection in innate immunity.	Endotoxin-like responses are triggered, leading to septic inflammation or immune tolerance.
**RKGKIHIPFPLANVTCMVPKA**	**Alpha-1B-glycoprotein**	Plasma glycoprotein, precise physiological function not well established.	Autoantibody generation may leads to systemic inflammation.
PTVTYGK	**ICAM-1**	Leukocyte adhesion and trafficking.	Autoimmunity against endothelium causes vasculitis and tissue infiltration.
**PTVTYGK**	**LRP2 (megalin)**	Endocytosis receptor in kidney and other tissues.	Autoantibodies cause immune-mediated nephritis.
CGTAEC	**IL-23 receptor**	Drives Th17 differentiation and inflammation.	Persistent Th17 activation results in autoimmune inflammation (psoriasis).

PDYSCKVFTGVYPFMWGGAYCFCD	**Platelet glycoprotein 4 (CD36)**	Class B scavenger receptor involved in uptake of oxidized lipids and fatty acids, platelet function, and innate immune sensing; roles in inflammation and thrombosis.	Autoantibodies promote vascular inflammation and thrombosis.
PDYSCKVFTGVYPFMWGGAYCFCD	**Glucocorticoid receptor**	Regulates inflammation and stress responses.	Inhibit receptor signaling resulting in uncontrolled inflammation.
PDYSCKVFTGVYPFMWGGAYCFCD	**Androgen receptor**	Regulates androgen-dependent signaling.	Cross-reactivity could disrupt androgen signaling leading to endocrineimmune imbalance.
FSTALAS	**LECT2**	Chemotactic factor for leukocyte recruitment.	Excess immune infiltration drives tissue injury.
PPCIPCCYEKEPEETLRMLEDNV	**IL-1RAP**	Amplifies IL-1 inflammatory signaling.	Hyperactive IL-1 signaling produces autoinflammatory disease.
**YSGGRFTIPTGAGKPGDSGRPIFDN**	**Collagen II (α1 chain)**	Cartilage structural protein.	Cross-reactive immunity could leads to autoimmune arthritis.
RKGKIHIPFPLANVTCMVPKA	**TRAF6**	Adapter for TNF/IL-1/TLR signaling leading to NF-κB activation.	NF-κB dysregulation drives chronic inflammation.
**YNMDYPPFGAGRPGQFGDIQSRTPE**	**Annexin A5**	Anti-coagulant phospholipid-binding protein.	Autoantibodies could cause antiphospholipid syndrome and thrombosis.
DIPDAAFTRVVDAP	**Vitronectin**	Regulates complement and clotting.	Viral mimicry may block complement attack and trigger vascular autoimmunity.

DGTLKIQVSLQIG	**CD2**	T-cell adhesion and costimulation.	Aberrant T-cell priming may initiates autoreactive responses.
TSAPCTITGTMGHFILARCPKG	**Integrin αIIb (GPIIb)**	Platelet integrin for aggregation.	Autoantibodies could destroy platelets resulting to immune thrombocytopenia.
KWQYNSPLVPR	**Leptin receptor (LEPR)**	Energy homeostasis and immune modulation.	Dysregulated signaling links obesity to chronic inflammation.
KWQYNSPLVPR	**PGLYRP1**	Recognizes bacterial peptidoglycan.	Pathogen sensing may be misdirected, driving autoimmunity.
DGTLKIQVSLQIG	**β-sarcoglycan**	Muscle structural protein in dystrophin complex.	Cross-reactivity may drive muscular dystrophy–like autoimmunity.
MKSDASKFTHEKPEGYYNWHHGAVQ	**IGSF6**	Leukocyte immunoglobulin superfamily receptor. precise role in humans is not well defined.	Dysregulated cell signaling activates inappropriate immune responses.
YSGGRFTIPTGAGKPGDSGRPIFDN	**Macrophage scavenger receptor I/II (MSR1)**	Clears modified LDL, pathogens, apoptotic cells.	Defective clearance and autoantibody production may cause chronic inflammation.
KFIVGPMSSAWTPFDNKIVVYKGDV	**Interleukin-23 receptor**		
SKDVYANTQLVLQRPAAGTVHVPYS	**FcγRI (CD64)**	High-affinity IgG receptor for phagocytosis.	Autoantibodies trigger excessive self-tissue clearance.
APFGCQIATNPVRAMNCAVGNMPIS	**Coagulation factor V**	Essential clotting factor.	Cross-reactive antibodies could disrupt clotting resulting to thrombosis or bleeding disorders.
RKGKIHIPFPLANVTCMVPKA	**MICB**	Stress-induced ligands for NK/T cell activation.	Viral mimicry may impair NK surveillance or drive autoimmunity.

KFIVGPMSSAWTPFDNKIVVYKGDV	**LY75 (DEC-205)**	Antigen uptake receptors on dendritic cells.	Defective antigen presentation could impair tolerance.
KPGRRERMCMKIENDCIFEVK	**Protocadherin-9**	Neuronal adhesion and synaptic organization.	Autoimmunity could cause neuroinflammation and synaptic dysfunction.
**SKDVYANTQLVLQRPAAGTVHVPYS**	**CILP (Cartilage intermediate layer protein 1)**	ECM cartilage protein, regulates TGF-β signaling.	Autoantibodies could cause cartilage destruction and arthritis.
FIPTQTFY	**Complement C3**	Central complement protein in opsonization and inflammation.	Complement dysregulation drives autoimmune tissue damage.
CGTAEC	**Integrin αV**	Adhesion, angiogenesis, ECM remodeling.	Autoantibodies may cause endothelial and vascular inflammation.
RKGKIHIPFPLANVTCMVPKA	**LFA-3 (CD58)**	Ligand for CD2, activates T cells.	T-cell activation is distorted, may cause autoreactive T-cell responses.
NADLAKLAFKRSSKYDLECAQIPVH	**TNF receptor-associated factor 6**		
FIPTQTFY	**FcεRIα**	High-affinity receptor for IgE on mast cells.	May cause allergic-type autoimmunity and mast cell activation
PGYYQLL	**NAT16**	Acetylates proteins.	-
PTVTYGK	**VSIG4**	Complement receptor and T-cell inhibitor	Viral mimicry may suppress T-cell responses, enabling viral persistence.
RKGKIHIPFPLANVTCMVPKA	**TLR7**	Recognizes viral ssRNA, induces IFN-I.	Overactivation may drive autoimmunity.

**EGLEVTWGNN**	**CHI3L2 (Chitinase-3-like protein 2)**	ECM remodeling and tissue repair.	Autoantibodies could promote chronic inflammation.

Hogeboom 2015 - Peptide motif analysis predicts alphaviruses as triggers for rheumatoid arthritis				
Virus	Viral peptide	Human protein	Normal function **(UniProt)**	Potential Viral Mimicry Impact on Immune Response
SFV	VHMKSDASKYTHE	Collagen II	Cartilage structural protein.	Cross-reactive immunity leads to autoimmune arthritis.
MAYV	VHMKSDASKYTHE			
ONNV	VHMKSDASKYFHE			
CHIKV	VHMKSDASKYFHE			
BFV	VCMKSDASKYFHE			
SINV	VNMRSEAFTYTSE			

Reddy et.al., 2017 - Molecular Mimicry between Chikungunya Virus and Host Components: A Possible Mechanism for the Arthritic Manifestations				
Virus	Viral peptides	Protein	Function **(UniProt)**	Potential Viral Mimicry Impact on Immune Response
CHIKV	Peptide A: GDIQSRTPESKDVYANTQLV<br>Peptide B: IKYAVSKKGKCAVHSMTNAV<br>Peptide C: TAECKDKNLPDYSCKVFTGV	hsDDB1–hsDDB2 complex (DNA damage-binding proteins)	Recognizes UV-damaged DNA, initiates nucleotide excision repair.	Distorted DNA repair responses could activate immunity against nuclear proteins
		Complement component C3	Central complement protein in innate immunity; opsonization,	May trigger uncontrolled complement activation and tissue injury.

	Peptide D: QLSEAHVEKSESCKTEFASAY		inflammation, MAC activation.	
		Fibronectin	Extracellular matrix glycoprotein; cell adhesion, wound healing.	Could elicit autoantibodies driving connective tissue autoimmunity.
		Immunoglobulin G (IgG)	Central adaptive immunity effector; neutralization, opsonization, complement activation.	Induces anti-IgG autoantibodies (rheumatoid factor–like autoimmunity).
		Kelch-like protein	Scaffold protein; regulates ubiquitination and oxidative stress.	Could disrupt protein degradation leading to accumulation of self-antigens and autoimmunity.
		β-arrestin 1	Adapter protein for GPCR trafficking; binds clathrin.	Could disrupt receptor desensitization, sustaining immune receptor signaling leading to chronic inflammation.
		Mast/stem cell growth factor receptor (KIT)	Controls mast cell survival, hematopoiesis.	Drives uncontrolled mast cell activation leading to allergy-like autoimmunity.
		Complement component C5	Complement activation protein; drives C5a inflammation and MAC formation.	May over activates complement cascade leading to severe inflammatory damage.
		Voltage-dependent anion channel protein 1 (VDAC1)	Mitochondrial porin; regulates ATP transport and apoptosis.	Could Triggers anti-mitochondrial autoimmunity and metabolic dysfunction.

		Vacuolar protein sorting 26 (VPS26)	Part of retromer complex; endosomal trafficking; has arrestin fold.	Could Interferes with antigen recycling resulting to abnormal antigen presentation and autoimmunity.
		Platelet-activating factor acetylhydrolase IB α subunit	Regulates inflammatory lipid mediator PAF.	Skews lipid-mediated immunity
		ARP2/3 complex subunit 4	Initiates polymerization; regulates cell motility and phagocytosis.	Disrupts actin remodeling resulting to defective immune synapse or cytotoxic killing.
		Interleukin-13 receptor α1	IL-13 signaling; drives Th2-type immunity.	May Induces Chronic Th2 bias
		Antibody Fab fragment	Antigen recognition region of an antibody.	Could induce cross-reactive antibodies resulting in autoimmunity against host antigens.
		PALB2 (WD40 domain)	Partners with BRCA2 in DNA repair.	Drives anti-DNA repair autoantibodies
		Receptor protein tyrosine phosphatase	Regulates receptor tyrosine kinase signaling.	Dysregulates T-cell activation thresholds → inappropriate immune activation.
		Antibody YTS 105.18 (anti-CD8α)	Blocks CD8+ T cell activation.	Suppresses CD8+ T cell function leading to impaired viral clearance and autoimmunity.
		Laminin α subunit	Basement membrane protein; tissue structure, adhesion.	Drives anti-basement membrane autoimmunity
		Contactin-2	Neural cell adhesion; axon organization.	Induces neuronal autoimmunity

Ding et.al., 2021 - Chikungunya virus and autoimmunity: Consensus immune epitope analysis between Chikungunya virus and arthritis				
CHIKV	Viral peptides	Human protein	Function<br><br>**(UniProt)**	Potential Viral Mimicry Impact on Immune Response
NSP1	AFSHL	Cd97 antigen	Adhesion GPCR on leukocytes; regulates cell migration and immune activation.	Altered leukocyte trafficking may drive inappropriate inflammation
	DPERL	C3 complement	Central opsonin in complement cascade; mediates pathogen opsonization and inflammation.	Dysregulates complement and induces anti-C3 autoantibodies.
	RGKLS	Protein-arginine deiminase type-4	Citrullinates proteins, regulates chromatin and neutrophil extracellular traps.	May break tolerance to citrullinated proteins, leading to RA-like disease.
	SVFHL	Macrophage colony stimulating factor 1	Growth factor controlling macrophage survival and differentiation.	Skews macrophage polarization toward pathogenic inflammatory states.
	IVVNG	Inter-alpha trypsin inhibitory heavy chain H2	Stabilizes extracellular matrix via hyaluronan binding.	ECM destabilization may expose hidden epitopes, provoking autoimmunity
	AKECR	Proteasome subunit beta type-8	Immunoproteasome subunit; shapes antigen peptides for MHC-I.	Alters peptide presentation and may activates autoreactive CD8 T-cells.
	DEKLL	Fc receptor-like protein3	Immunoregulatory receptor on B/T cells; modulates activation.	Disruption of tolerance may promote autoantibody formation.
	EKLLG	Signal transducer and activator of transcription 3	Regulates gene expression for cell survival, proliferation, differentiation, and immune responses, including Th17/Treg balance	Alters cytokine signaling, skews T-cell differentiation, and drives chronic inflammation or autoimmune responses.
	QEDVA	Centrosome-associated protein CEP250	Maintains centriole cohesion.	May triggers anti-centrosome autoantibodies linked to inflammation.

NSP2	GDLVL	Endoplasmic reticulum chaperon BiP	ER chaperone, stress sensor, protein folding.	Externalization may elicit autoantibodies.
	LVWKTL	Interleukin-6 receptor subunit beta	Shared receptor subunit for IL-6 family cytokines.	Abnormal signaling may fuel chronic inflammation.
	KLNDR	Transferrin receptor protein 1	Mediates cellular iron uptake.	Autoantibodies may disrupt iron metabolism.
	KAYSP	Lymphotoxin-alpha	TNF-family cytokine, lymphoid organogenesis, inflammation.	May induce anti-cytokine autoantibodies and hyperinflammatory responses
	PEVAL	Coatomer subunit alpha	COPI coat protein for ER–Golgi trafficking.	May induce ER stress.
	IIPAN	Tyrosin- protein kinase CSK	Src kinase negative regulator in TCR/BCR signaling.	-
NSP3	PRGLPG	Collagen alpha-1 (II) chain	Cartilage structural protein.	Induces cross-reactive arthritis
	TVPGTI	Aggrecan core protein	Major cartilage proteoglycan.	May induce anti-cartilage antibodies and post-viral arthritis.
	QVELL	ADAMTS5	Aggrecanase degrading cartilage ECM.	Exposes cartilage neo-epitopes and promotes autoimmunity.
	KQTEA	Pyrin	Inflammasome sensor controlling IL-1β release.	Activates inflammasome pathways and drives autoinflammation.
	EGNIT	MHC class I polypeptide-related sequence B	Stress-induced ligand for NKG2D on NK/T cells.	Subverts immune surveillance and induces anti-MIC autoantibodies.
	APPST	E3 ubiquitin protein ligase synoviolin	ER E3 ubiquitin ligase in protein quality control.	Provokes anti-ER autoantibodies, especially in synovium.
	TDDEL	PIK3CD	Catalytic subunit of PI3Kδ in leukocyte signaling.	Alters lymphocyte activation thresholds and drives autoreactivity.

NSP4	IIQRL	Serine/threonine-protein phosphatase PP1-gamma catalytic subunit	Broad Ser/Thr phosphatase regulating signaling.	Retunes phosphorylation and triggers anti-phosphatase responses.
	PTVSS	ADAM15	Metalloprotease in adhesion and shedding.	Dysregulates leukocyte–endothelial interactions and sheds autoantigens.
	SAVPSP	Glutamate decarboxylase 2	Enzyme for GABA synthesis in neurons/islets.	Induces T1D-related anti-GAD autoimmunity.
	KLGKPL	Vascular endothelial growth factor receptor 2, tyrosine-protein kinase	Endothelial VEGF receptor driving angiogenesis.	Induces vasculitis through anti-receptor autoantibodies.
	ELEKA	Protein S100-A8	Calcium-binding DAMP in neutrophils (calprotectin).	Amplifies PRR signaling and drives auto-inflammation.
E2	LERIR	DNA- directed RNA polymerase III subunit RPC1	RNA polymerase III subunit, innate DNA sensor.	Induces anti-Pol III autoantibodies and interferonopathy.
	PKGET	High mobility group protein B1	Chromatin-binding protein, extracellular DAMP.	Induces chronic alarmin signaling and anti-HMGB1 antibodies.
	KEVVL	Aggrecan core protein	Major cartilage proteoglycan.	May induce anti-cartilage antibodies and post-viral arthritis.
	EGLEV	Ribonuclease H3 subunit C2	IFN-induced antiviral effector protein.	Derails antiviral defense and induces anti-IFIT autoantibodies.
	VTWGN	Interferon-induced protein with tetratricopeptide repeats 3	Inhibitor of calpains (cytoskeletal proteases).	Unleashes calpain activity and generates autoantigens.
Capsid	AGKPGD	Collage alpha-1(II) chain	Cartilage structural protein	
	GKPGD	Calpastatin	Endogenous calpain inhibitor	Dysregulates protease balance and exposes autoantigens.

	ARTAL	Tyrosine-protein kinase receptor Tie-1	Endothelial receptor tyrosine kinase	
E1	NITVT	Vascular endothelial growth factor receptor 1	Endothelial VEGF receptor/decoy.	Induces anti-endothelial autoantibodies.
		MHC class I polypeptide-related sequence A	Stress-induced ligands for NK/T cell activation.	Viral mimicry may impair NK surveillance or drive autoimmunity.
		MHC class I polypeptide-related sequence B	Stress-induced ligand for NKG2D on NK/T cells.	Subverts immune surveillance and induces anti-MIC autoantibodies.
	RTPES	Tyrosine-protein phosphatase non-receptor type 22	Phosphatase regulating TCR signaling thresholds.	Mis-tunes T-cell signaling and increases autoreactivity.
